# Supplementary figures and images for: Predicting drug sensitivity of cancer cells based on DNA methylation levels
Source: PLoS One. 2021 Sep 10;16(9):e0238757. doi: 10.1371/journal.pone.0238757 (PMC8432830; doi:10.1371/journal.pone.0238757)

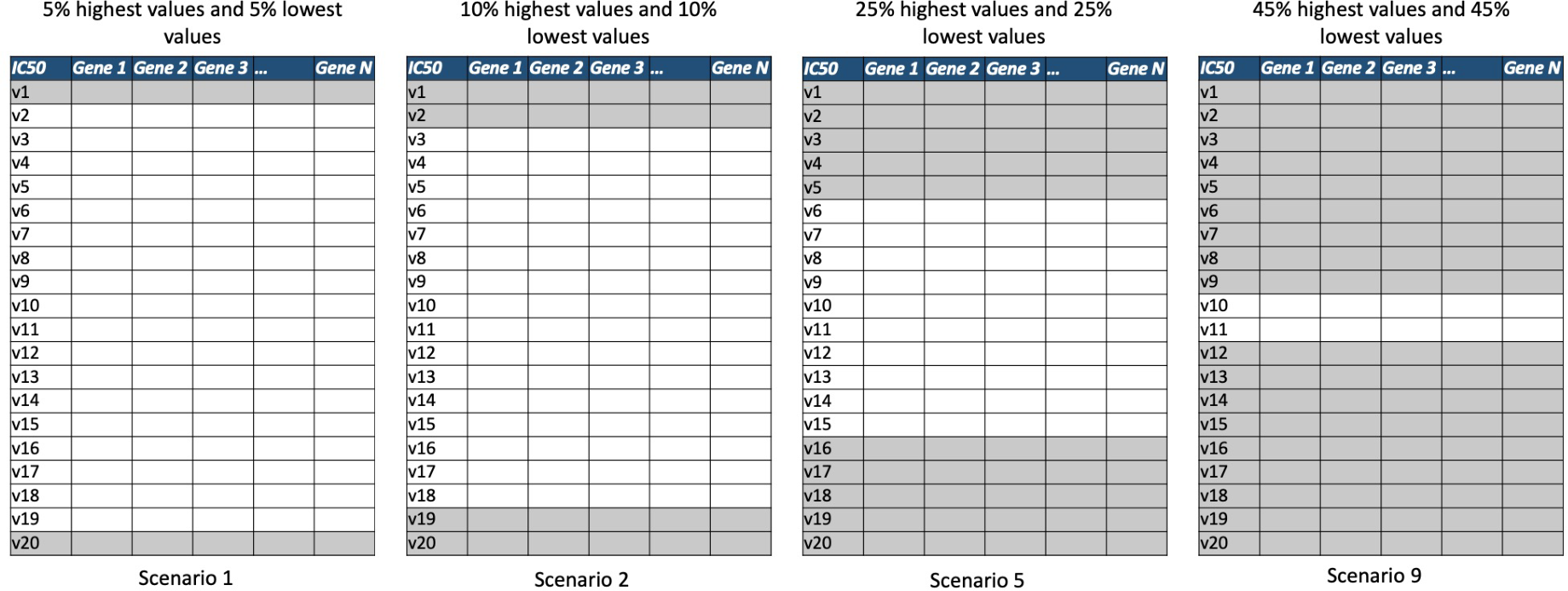

Supplement: S1 Fig — When performing classification, we discretized drug-response (IC50) values. To evaluate alternative thresholds for discretization, we performed a subsampling analysis. In Scenario 1 illustrated above, we considered the cell lines with the lowest and highest 5% of IC50 values. In Scenario 2, we considered the cell lines with the lowest and highest 10% of IC50 values. Each scenario used 10% more data than the previous scenario (5% on each side). This pattern continues until all data were considered in the analysis. (TIF) [file pone.0238757.s001.tif]

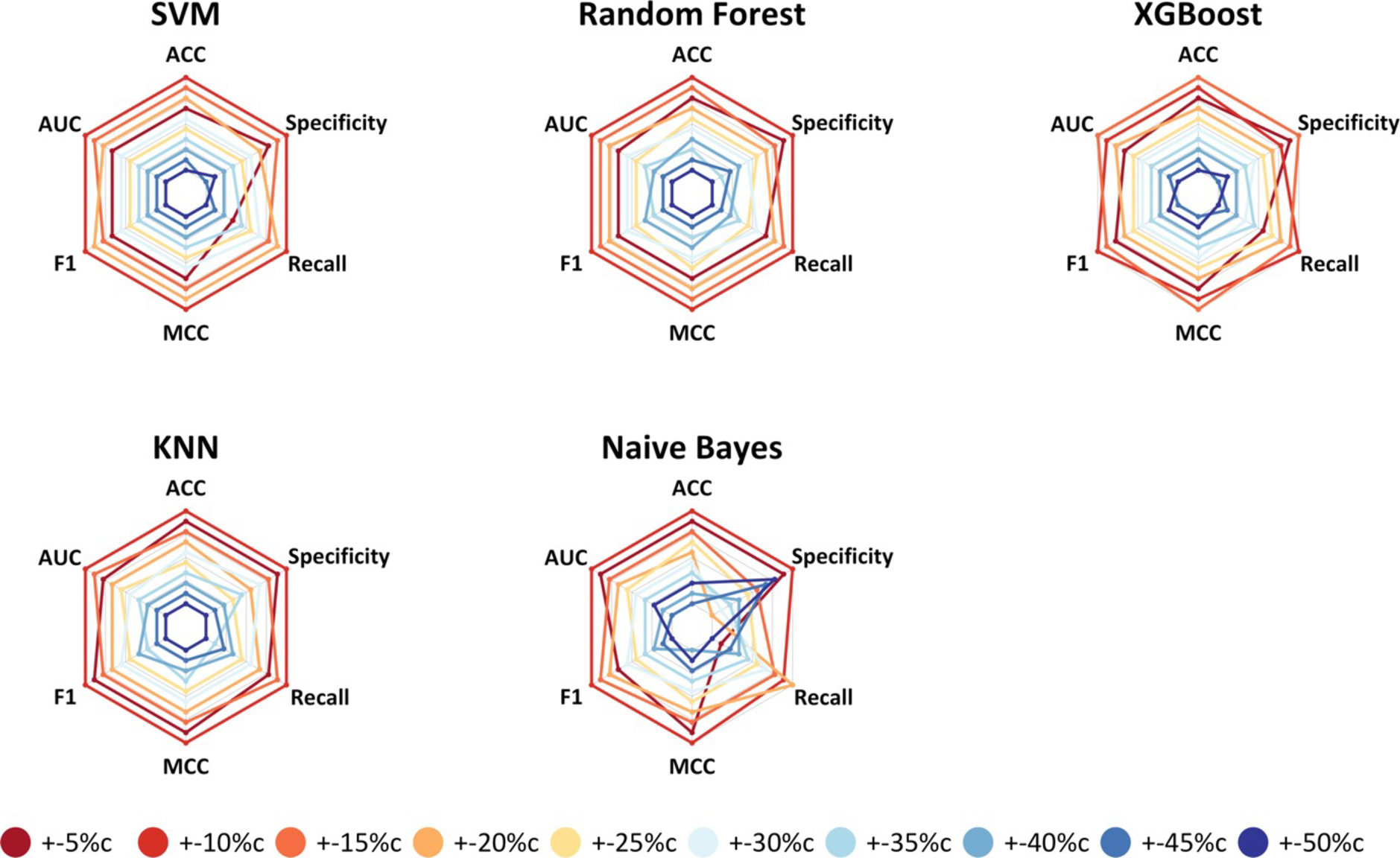

Supplement: S2 Fig — The graphs compare different scenarios ranked in order of best result. GDSC cell-line data were used to generate ten subsampling scenarios, which we then tested via nested cross validation. Scenarios that are further away from the center represent higher metric values than scenarios closer to it. The evaluated metrics for each algorithm are accuracy (ACC), specificity, recall, Matthews correlation coefficient (MCC), F1 score (F1) and area under the receiver operating characteristic curve (AUC). (TIF) [file pone.0238757.s002.tif]

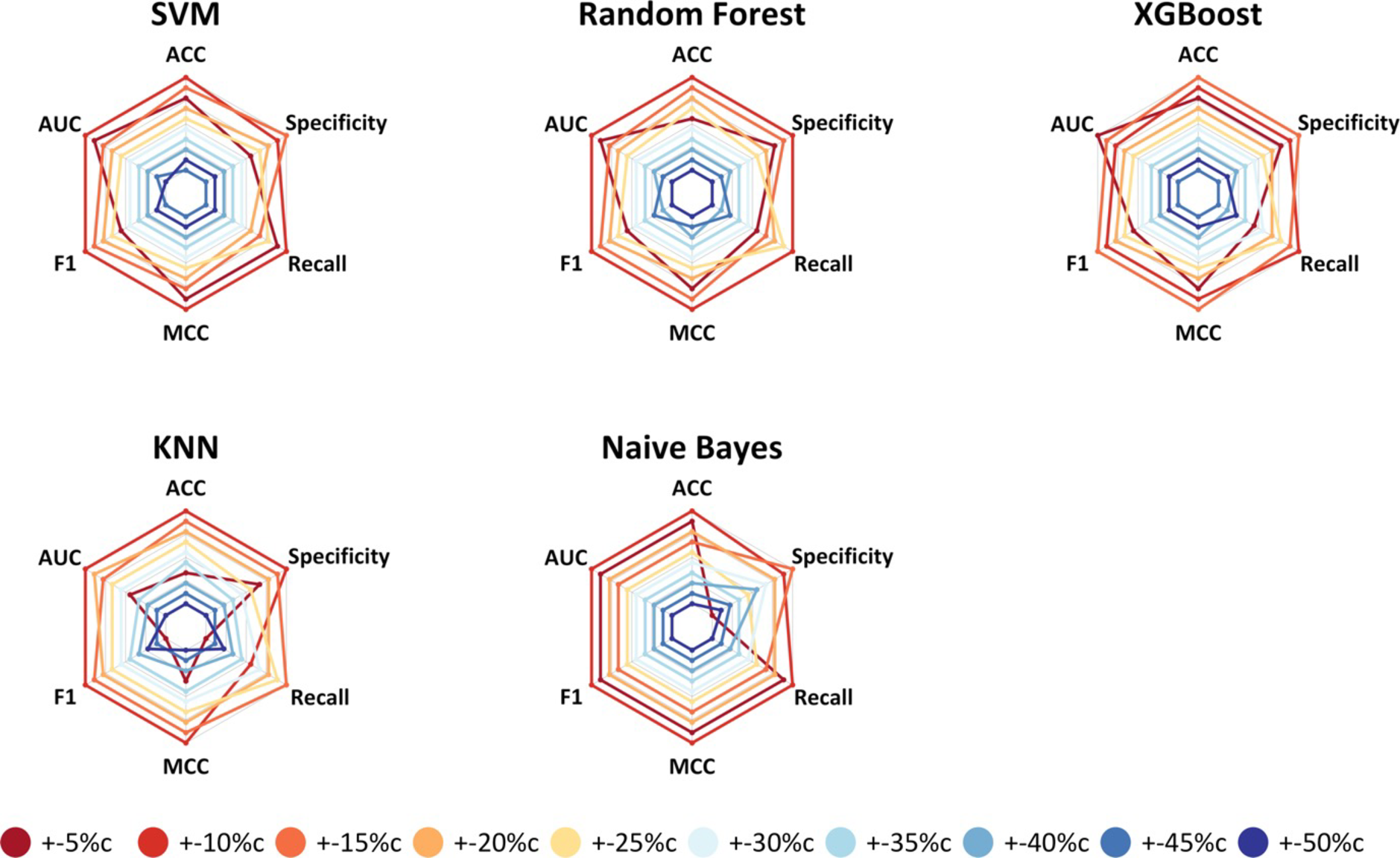

Supplement: S3 Fig — The graphs compare different scenarios ranked in order of best result. GDSC cell-line data were used to generate ten subsampling scenarios, which we then tested via nested cross validation. Scenarios that are further away from the center represent higher metric values than scenarios closer to it. The evaluated metrics for each algorithm are accuracy (ACC), specificity, recall, Matthews correlation coefficient (MCC), F1 score (F1) and area under the receiver operating characteristic curve (AUC). (TIF) [file pone.0238757.s003.tif]

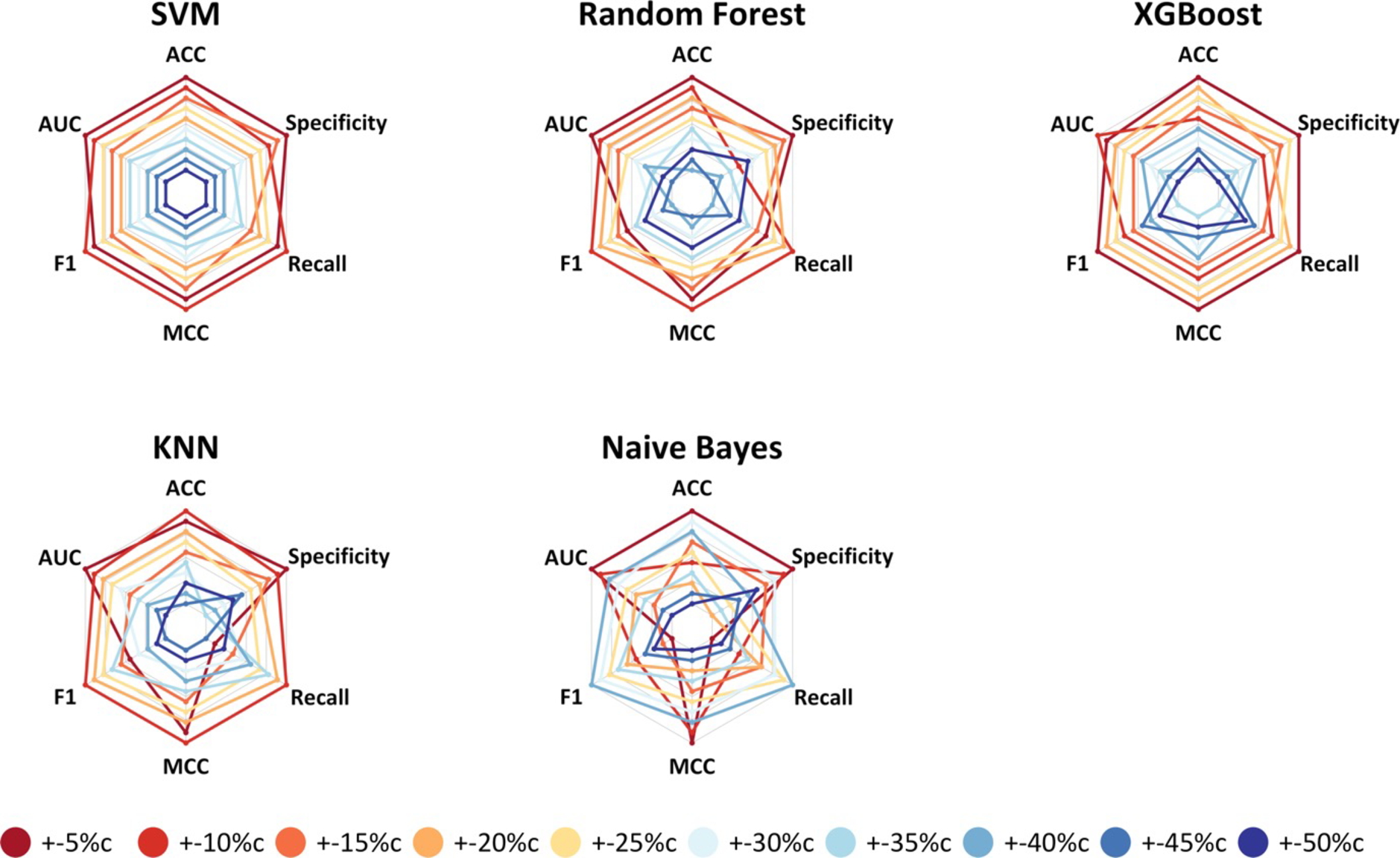

Supplement: S4 Fig — The graphs compare different scenarios ranked in order of best result. GDSC cell-line data were used to generate ten subsampling scenarios, which we then tested via nested cross validation. Scenarios that are further away from the center represent higher metric values than scenarios closer to it. The evaluated metrics for each algorithm are accuracy (ACC), specificity, recall, Matthews correlation coefficient (MCC), F1 score (F1) and area under the receiver operating characteristic curve (AUC). (TIF) [file pone.0238757.s004.tif]

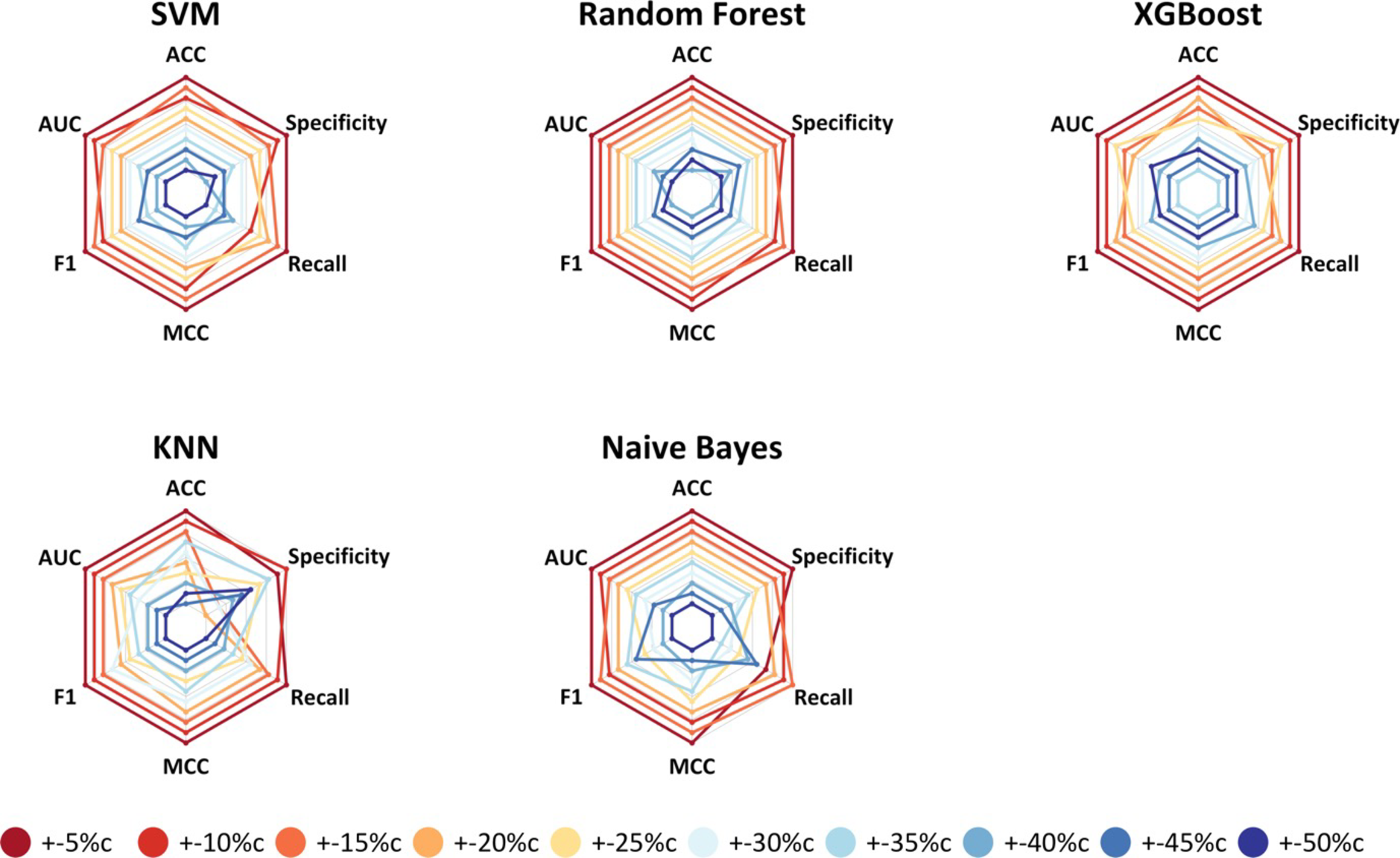

Supplement: S5 Fig — The graphs compare different scenarios ranked in order of best result. GDSC cell-line data were used to generate ten subsampling scenarios, which we then tested via nested cross validation. Scenarios that are further away from the center represent higher metric values than scenarios closer to it. The evaluated metrics for each algorithm are accuracy (ACC), specificity, recall, Matthews correlation coefficient (MCC), F1 score (F1) and area under the receiver operating characteristic curve (AUC). (TIF) [file pone.0238757.s005.tif]

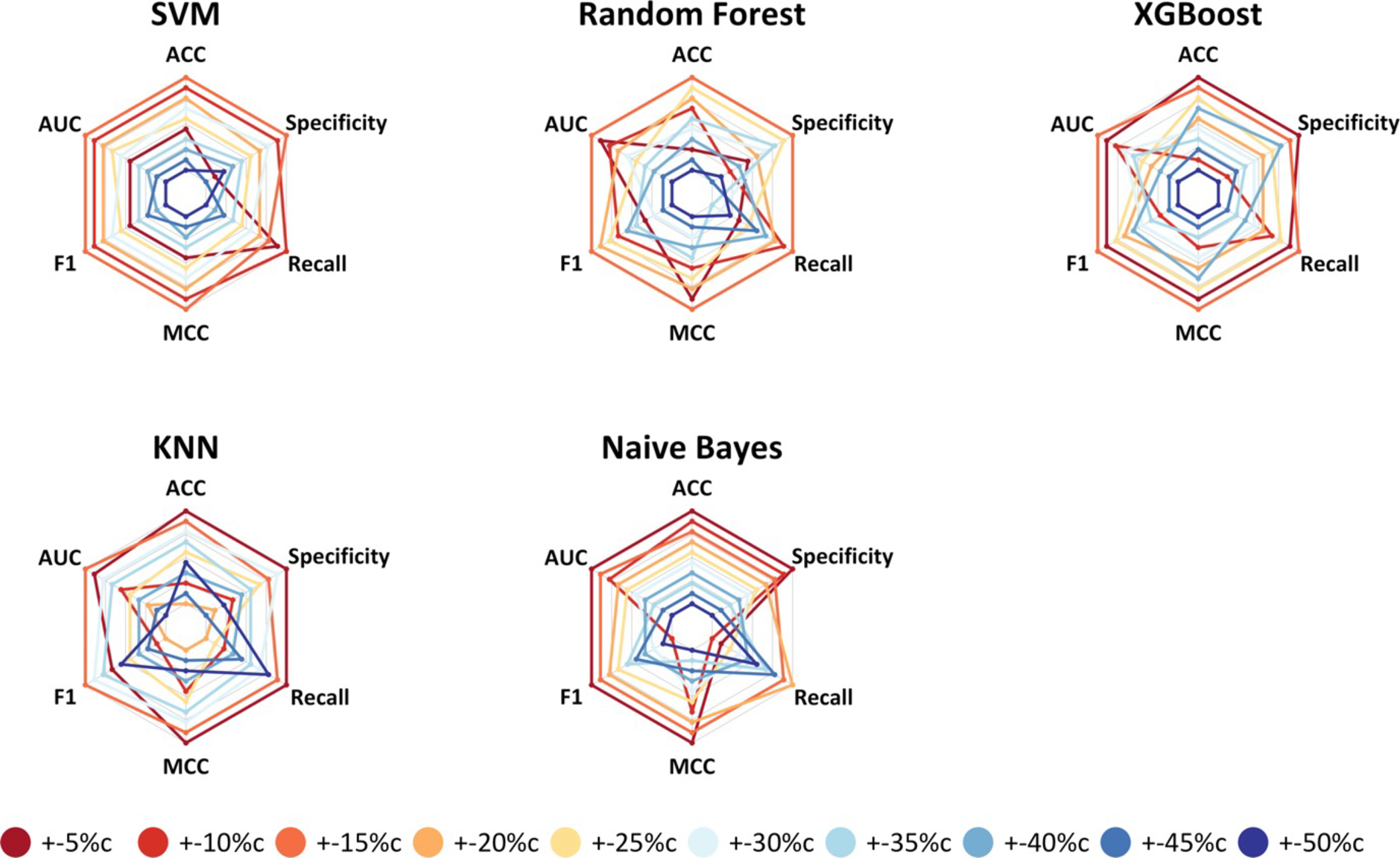

Supplement: S6 Fig — The graphs compare different scenarios ranked in order of best result. GDSC cell-line data were used to generate ten subsampling scenarios, which we then tested via nested cross validation. Scenarios that are further away from the center represent higher metric values than scenarios closer to it. The evaluated metrics for each algorithm are accuracy (ACC), specificity, recall, Matthews correlation coefficient (MCC), F1 score (F1) and area under the receiver operating characteristic curve (AUC). (TIF) [file pone.0238757.s006.tif]

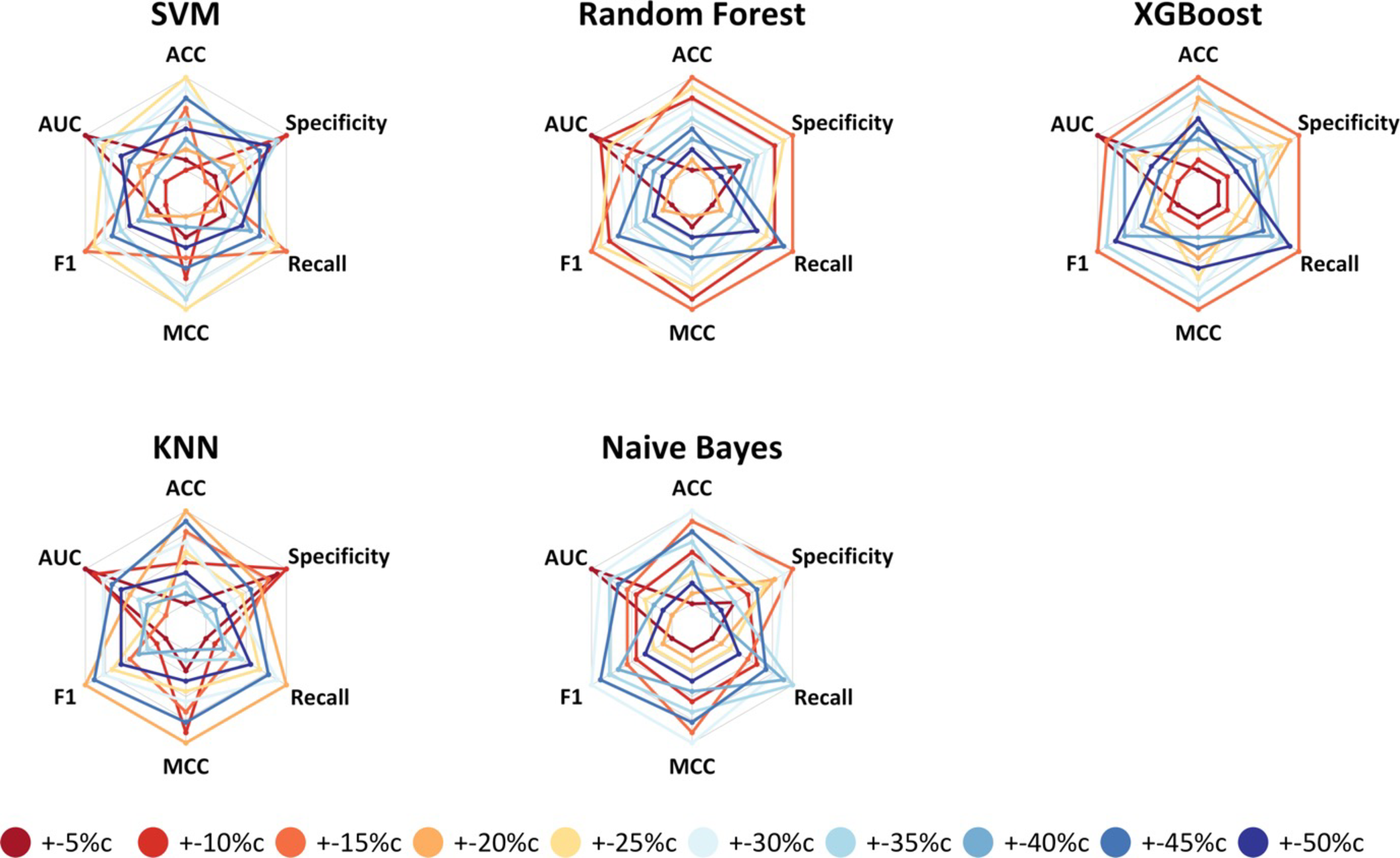

Supplement: S7 Fig — The graphs compare different scenarios ranked in order of best result. GDSC cell-line data were used to generate ten subsampling scenarios, which we then tested via nested cross validation. Scenarios that are further away from the center represent higher metric values than scenarios closer to it. The evaluated metrics for each algorithm are accuracy (ACC), specificity, recall, Matthews correlation coefficient (MCC), F1 score (F1) and area under the receiver operating characteristic curve (AUC). (TIF) [file pone.0238757.s007.tif]

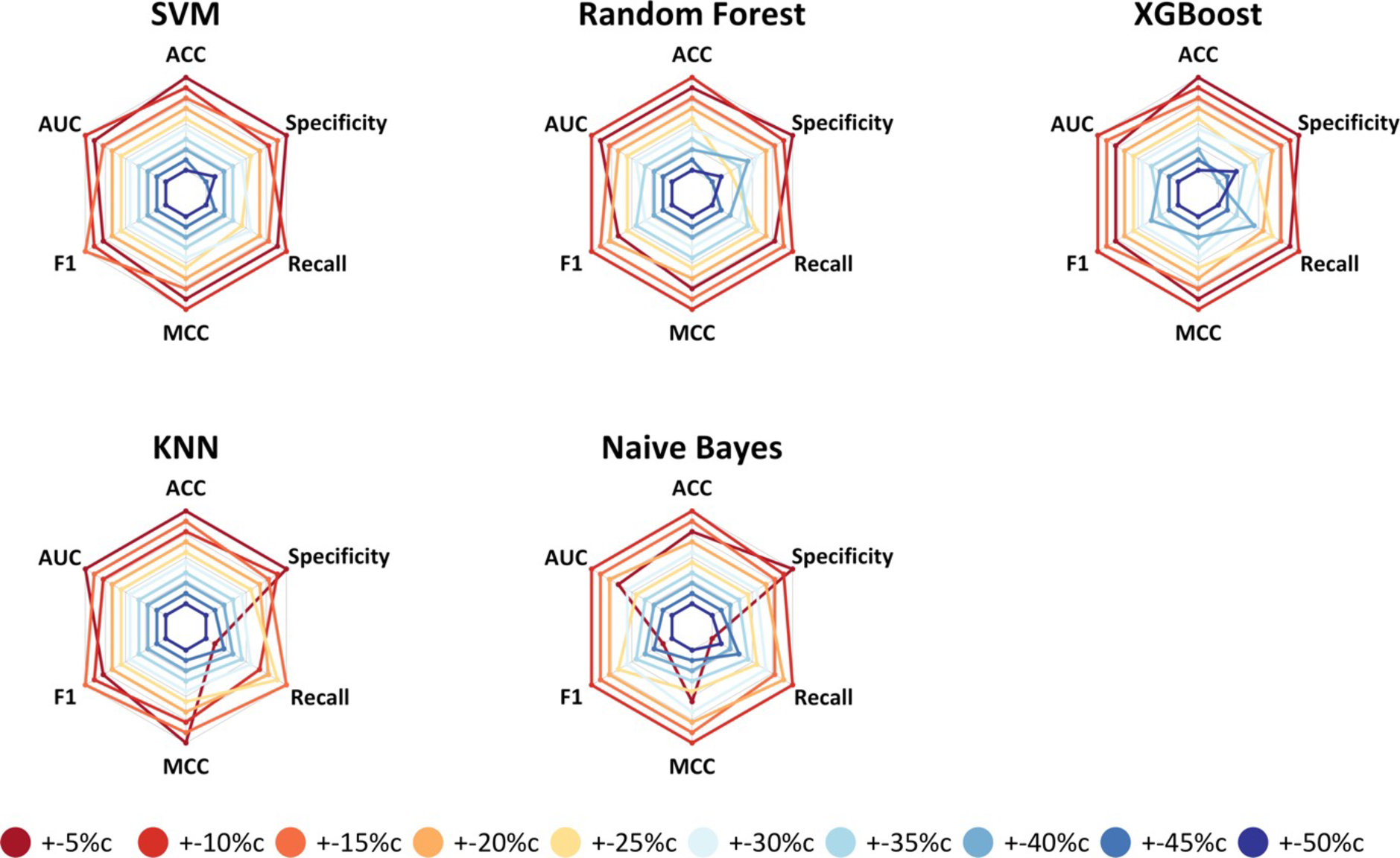

Supplement: S8 Fig — The graphs compare different scenarios ranked in order of best result. GDSC cell-line data were used to generate ten subsampling scenarios, which we then tested via nested cross validation. Scenarios that are further away from the center represent higher metric values than scenarios closer to it. The evaluated metrics for each algorithm are accuracy (ACC), specificity, recall, Matthews correlation coefficient (MCC), F1 score (F1) and area under the receiver operating characteristic curve (AUC). (TIF) [file pone.0238757.s008.tif]

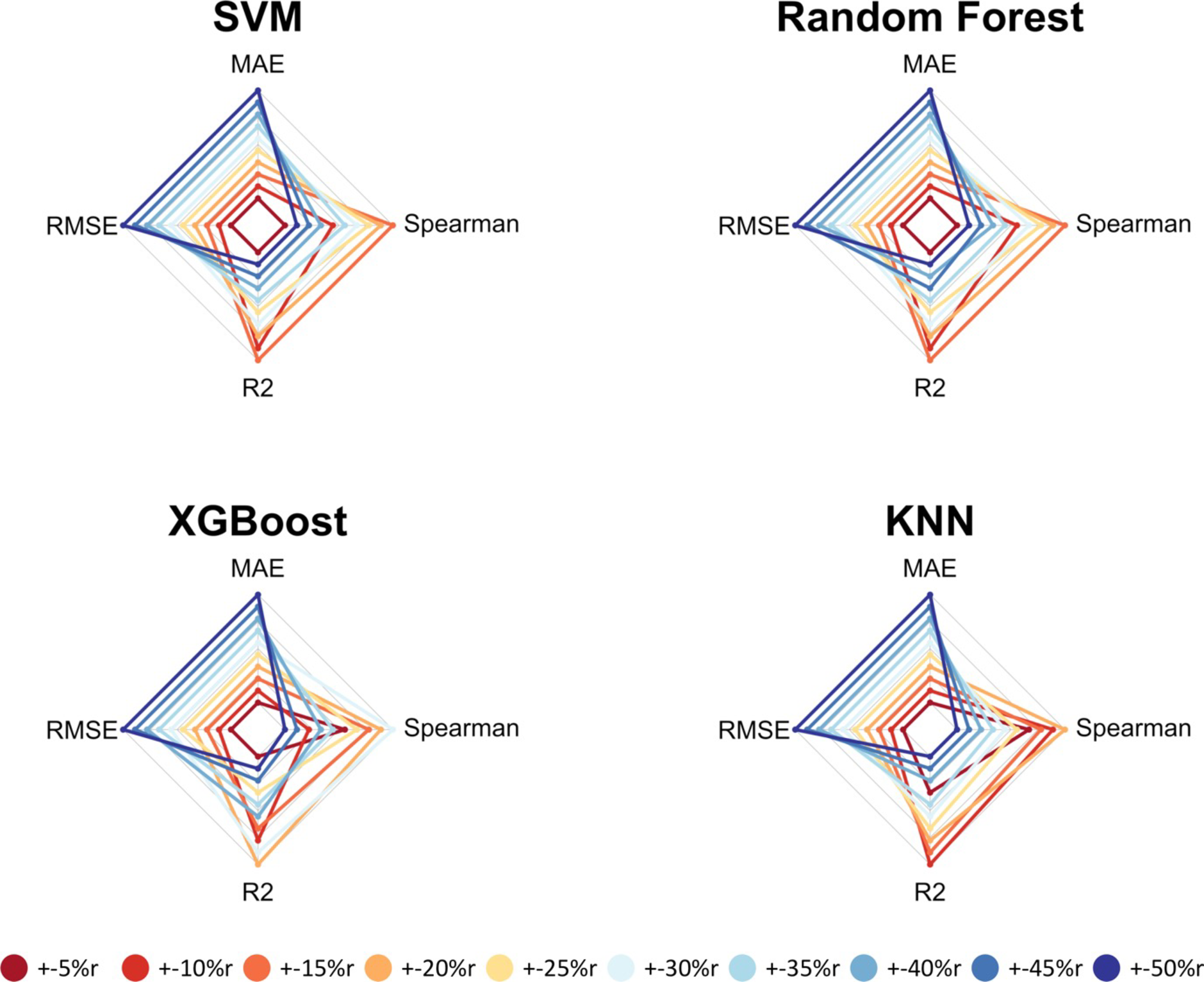

Supplement: S9 Fig — We used DNA methylation data from cell lines to predict continuous IC50 response values using four regression algorithms. We evaluated the algorithms’ performance via nested cross validation for ten subsampling scenarios. Graphs illustrate performance for these scenarios, ranked in order of relative performance for four metrics: RMSE (Root Mean Square Error), MAE (Mean Absolute Error), R-squared and Spearman correlation coefficient. Scenarios further away from the center represent relatively low metric values (and thus better performance). Scenarios that used all cell lines performed best for all algorithms. (TIF) [file pone.0238757.s009.tif]

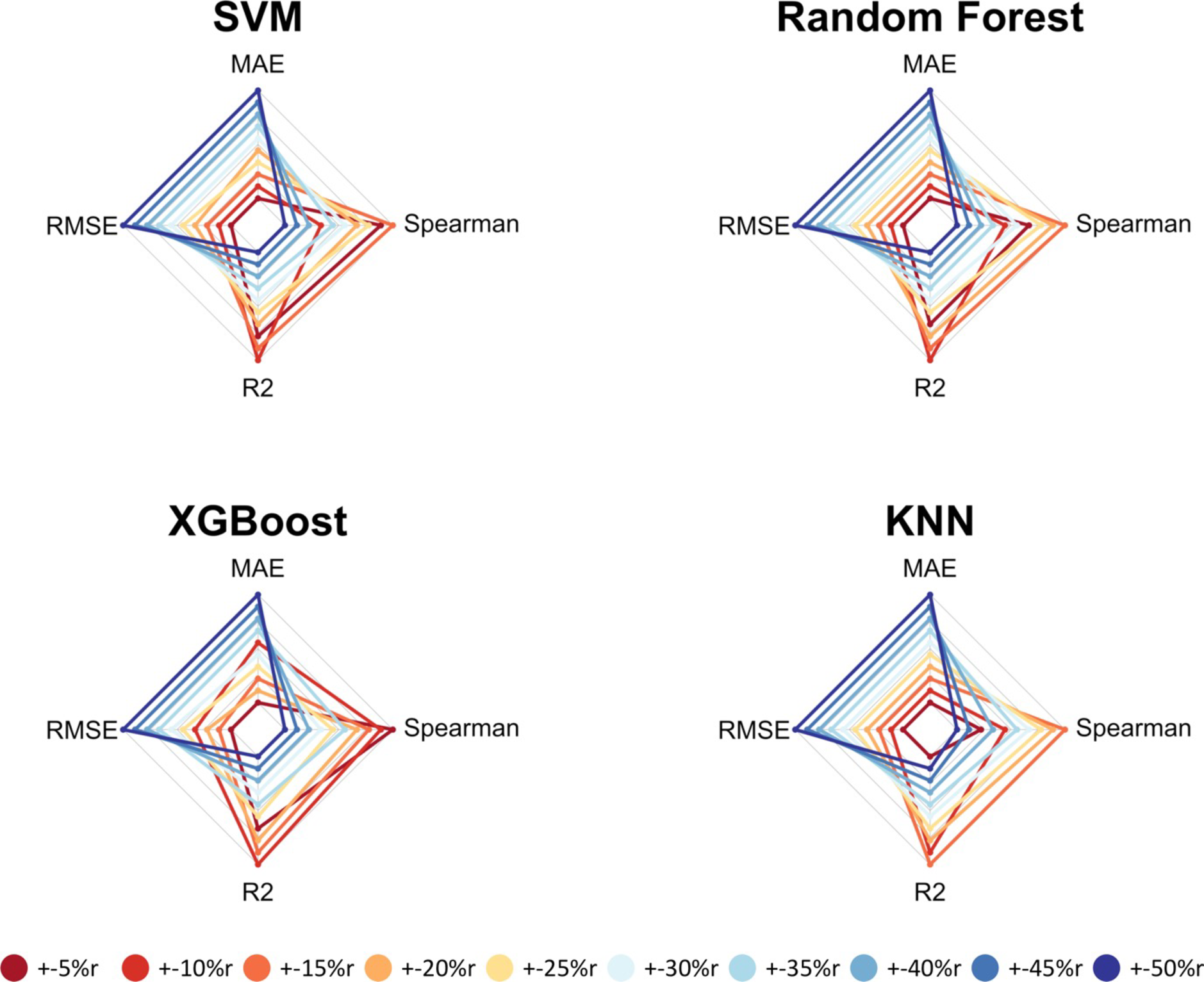

Supplement: S10 Fig — We used DNA methylation data from cell lines to predict continuous IC50 response values using four regression algorithms. We evaluated the algorithms’ performance via nested cross validation for ten subsampling scenarios. Graphs illustrate performance for these scenarios, ranked in order of relative performance for four metrics: RMSE (Root Mean Square Error), MAE (Mean Absolute Error), R-squared and Spearman correlation coefficient. Scenarios further away from the center represent relatively low metric values (and thus better performance). Scenarios that used all cell lines performed best for all algorithms. (TIF) [file pone.0238757.s010.tif]

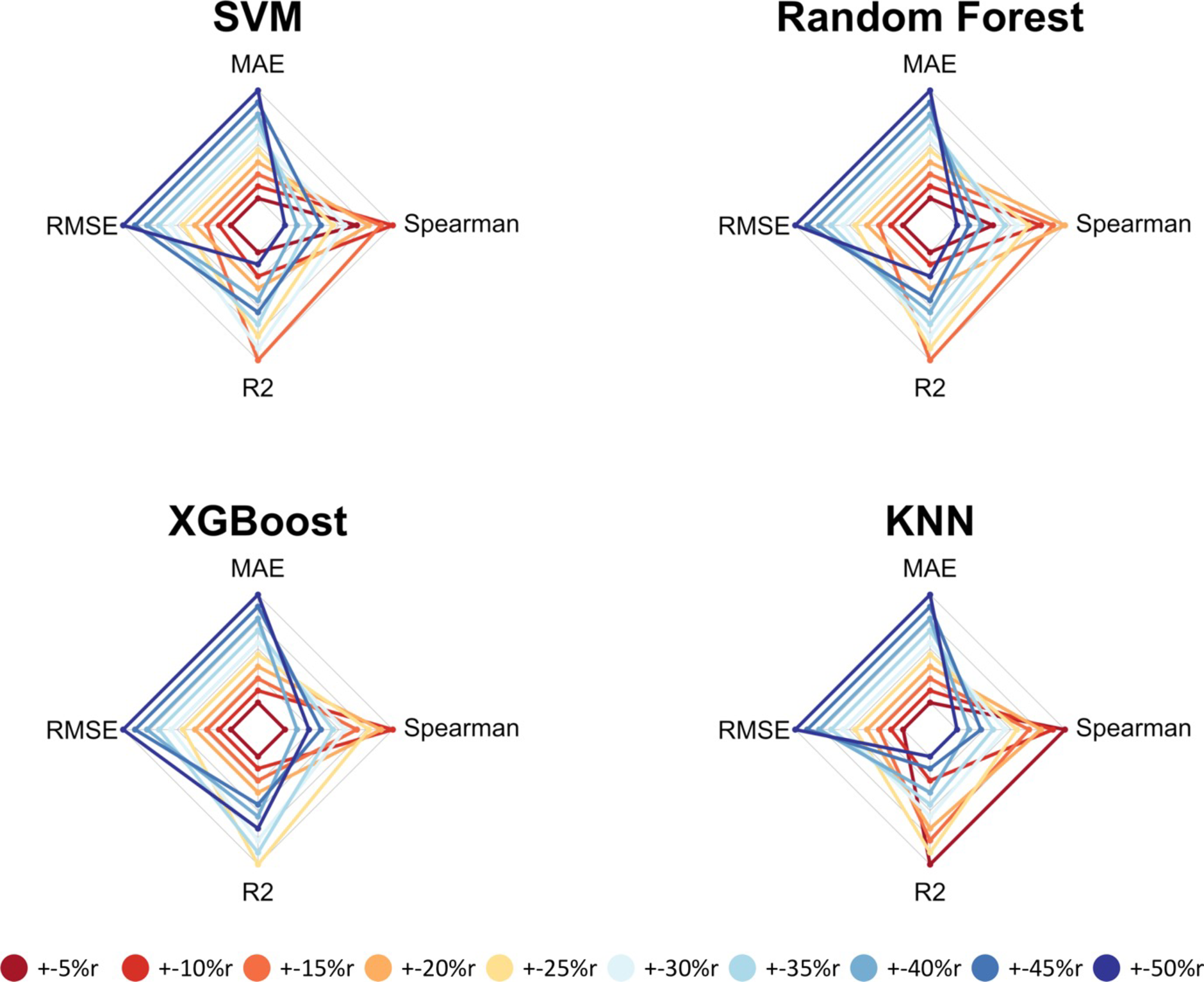

Supplement: S11 Fig — We used DNA methylation data from cell lines to predict continuous IC50 response values using four regression algorithms. We evaluated the algorithms’ performance via nested cross validation for ten subsampling scenarios. Graphs illustrate performance for these scenarios, ranked in order of relative performance for four metrics: RMSE (Root Mean Square Error), MAE (Mean Absolute Error), R-squared and Spearman correlation coefficient. Scenarios further away from the center represent relatively low metric values (and thus better performance). Scenarios that used all cell lines performed best for all algorithms. (TIF) [file pone.0238757.s011.tif]

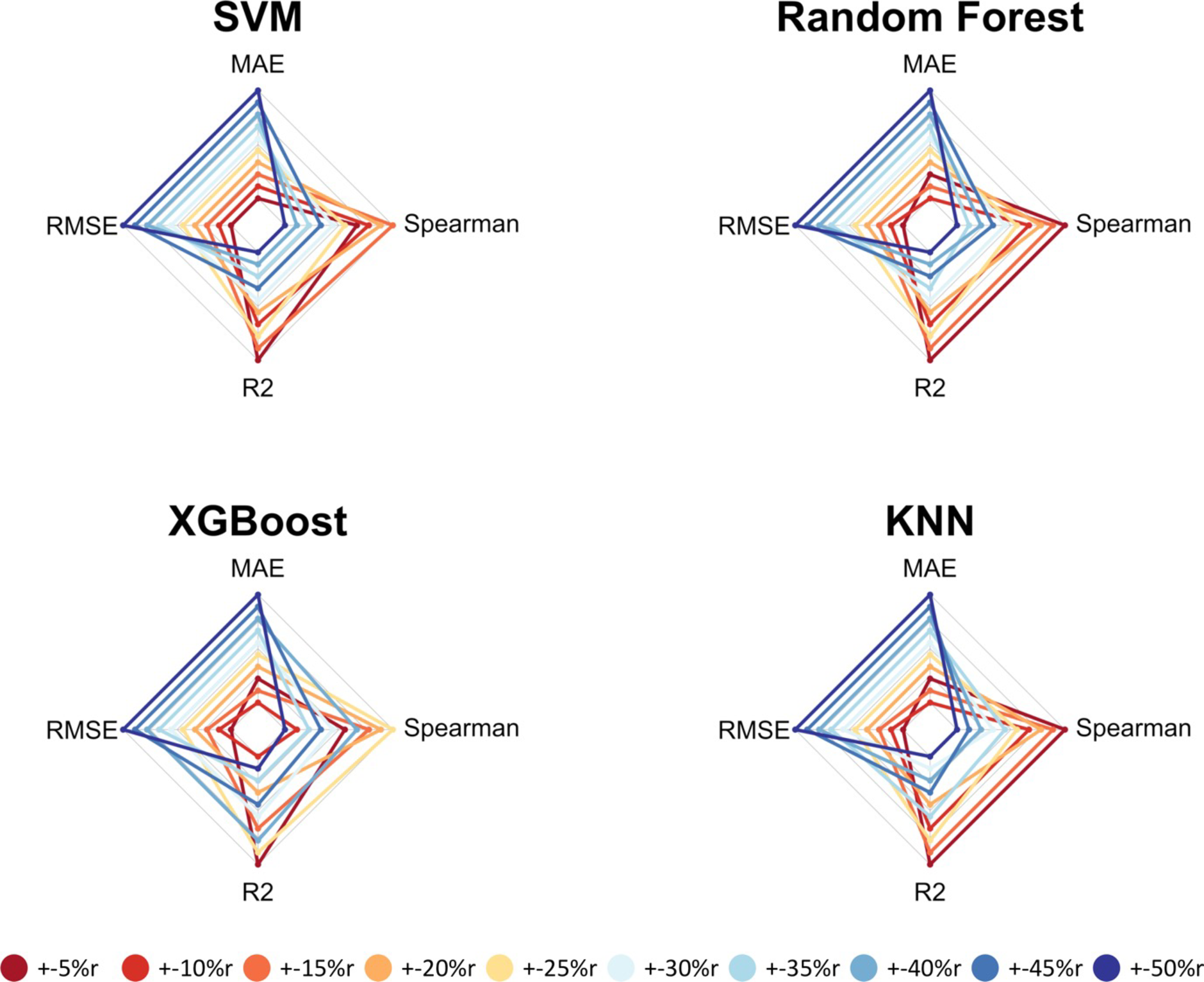

Supplement: S12 Fig — We used DNA methylation data from cell lines to predict continuous IC50 response values using four regression algorithms. We evaluated the algorithms’ performance via nested cross validation for ten subsampling scenarios. Graphs illustrate performance for these scenarios, ranked in order of relative performance for four metrics: RMSE (Root Mean Square Error), MAE (Mean Absolute Error), R-squared and Spearman correlation coefficient. Scenarios further away from the center represent relatively low metric values (and thus better performance). Scenarios that used all cell lines performed best for all algorithms. (TIF) [file pone.0238757.s012.tif]

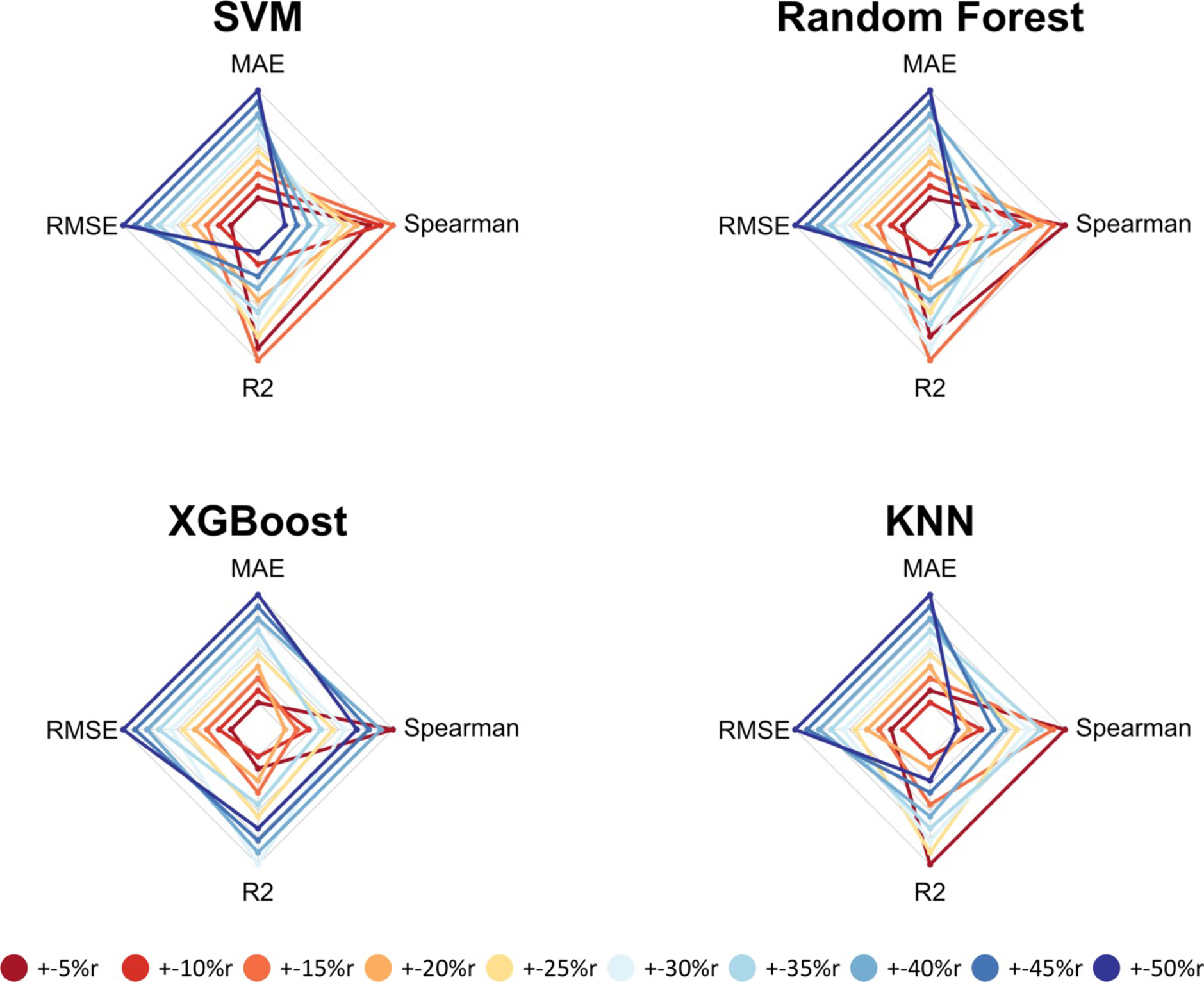

Supplement: S13 Fig — We used DNA methylation data from cell lines to predict continuous IC50 response values using four regression algorithms. We evaluated the algorithms’ performance via nested cross validation for ten subsampling scenarios. Graphs illustrate performance for these scenarios, ranked in order of relative performance for four metrics: RMSE (Root Mean Square Error), MAE (Mean Absolute Error), R-squared and Spearman correlation coefficient. Scenarios further away from the center represent relatively low metric values (and thus better performance). Scenarios that used all cell lines performed best for all algorithms. (TIF) [file pone.0238757.s013.tif]

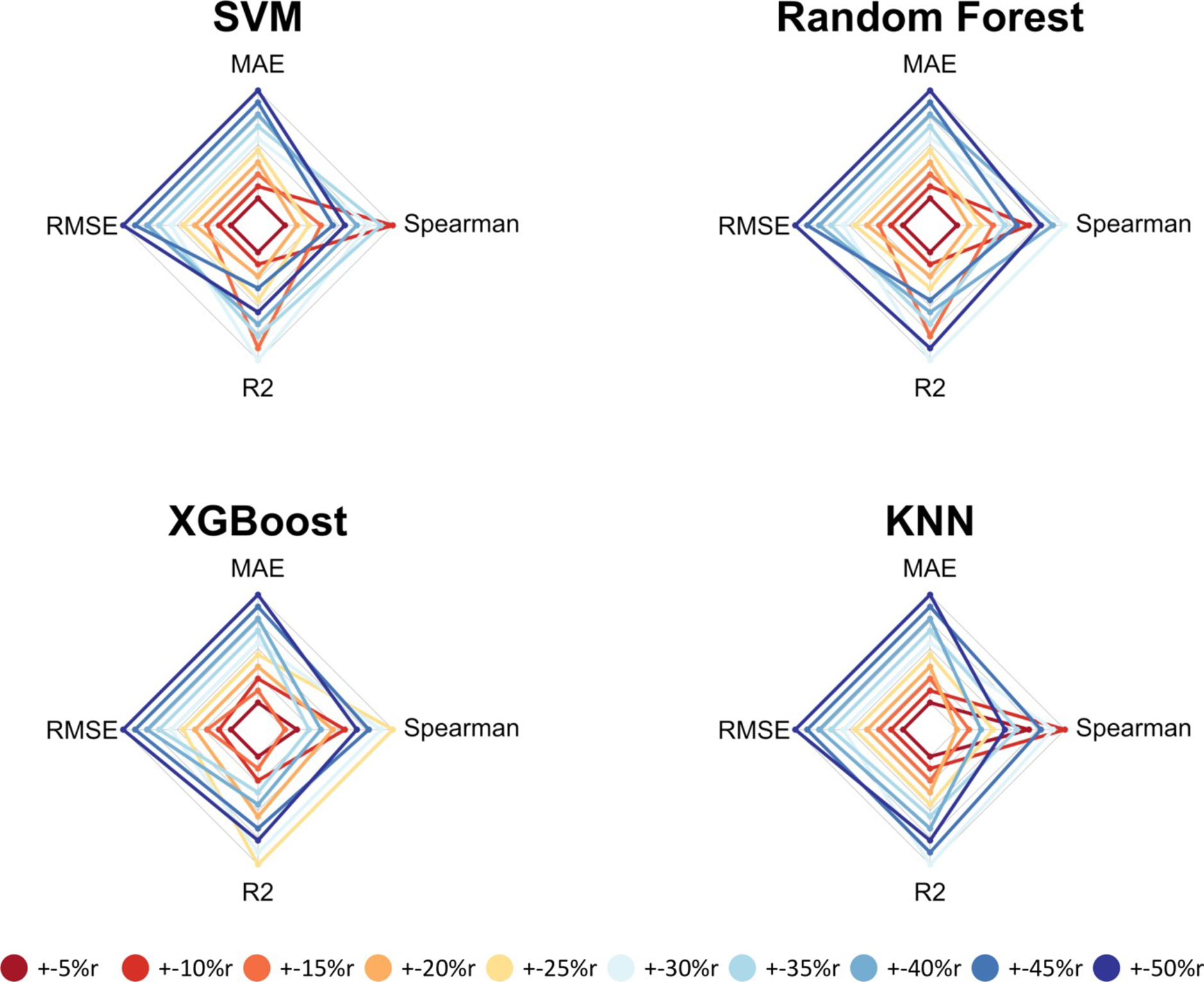

Supplement: S14 Fig — We used DNA methylation data from cell lines to predict continuous IC50 response values using four regression algorithms. We evaluated the algorithms’ performance via nested cross validation for ten subsampling scenarios. Graphs illustrate performance for these scenarios, ranked in order of relative performance for four metrics: RMSE (Root Mean Square Error), MAE (Mean Absolute Error), R-squared and Spearman correlation coefficient. Scenarios further away from the center represent relatively low metric values (and thus better performance). Scenarios that used all cell lines performed best for all algorithms. (TIF) [file pone.0238757.s014.tif]

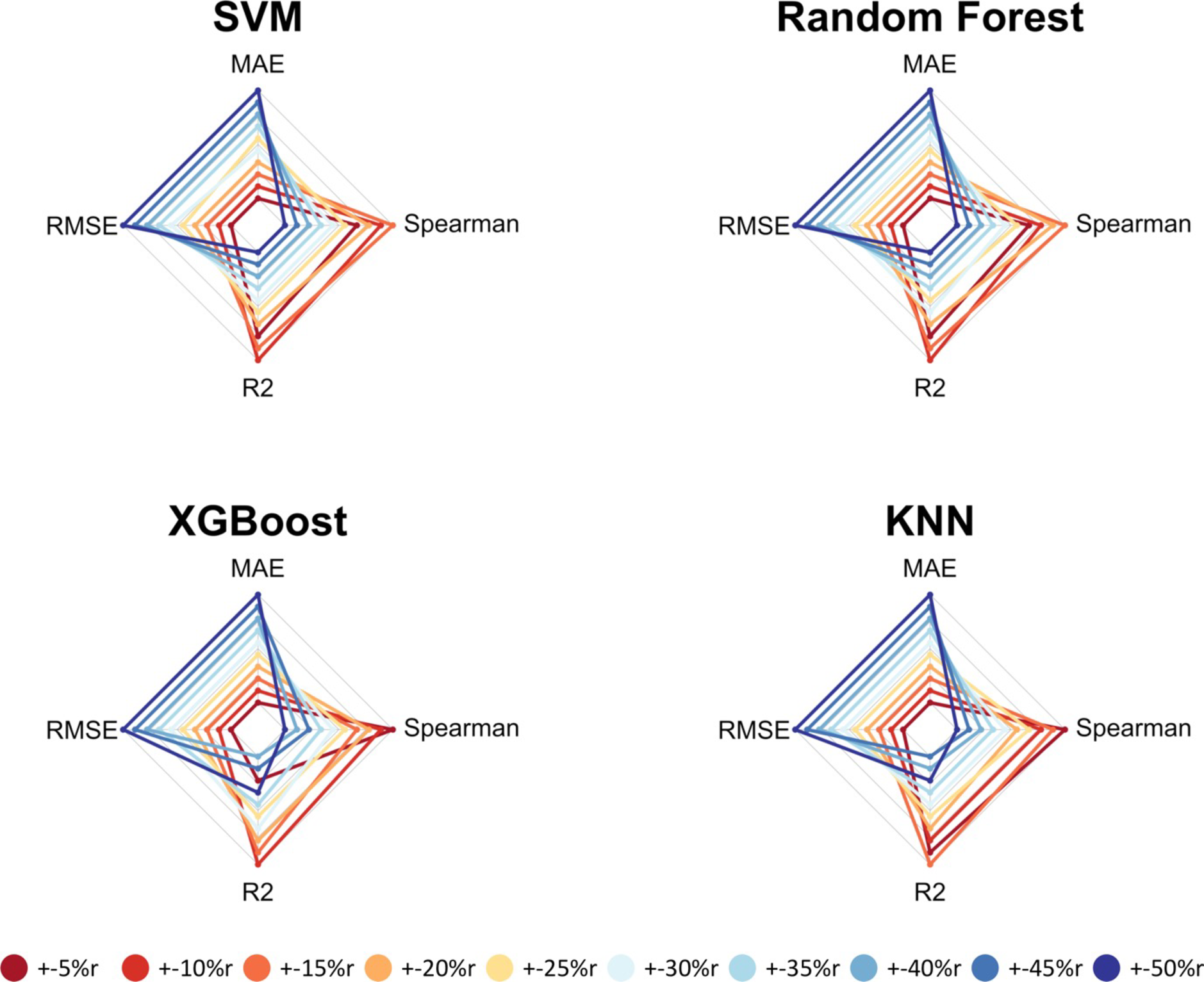

Supplement: S15 Fig — We used DNA methylation data from cell lines to predict continuous IC50 response values using four regression algorithms. We evaluated the algorithms’ performance via nested cross validation for ten subsampling scenarios. Graphs illustrate performance for these scenarios, ranked in order of relative performance for four metrics: RMSE (Root Mean Square Error), MAE (Mean Absolute Error), R-squared and Spearman correlation coefficient. Scenarios further away from the center represent relatively low metric values (and thus better performance). Scenarios that used all cell lines performed best for all algorithms. (TIF) [file pone.0238757.s015.tif]
