# Supplementary material for: Predicting drug sensitivity of cancer cells based on DNA methylation levels
Source: PLoS One. 2021 Sep 10;16(9):e0238757. doi: 10.1371/journal.pone.0238757 (PMC8432830; doi:10.1371/journal.pone.0238757)
Supplement: S5 Table — Bold font indicates the best-performing combination for each metric. (DOCX) [file pone.0238757.s020.docx]

| **Scenario** | **Method** | **ACC** | **AUC** | **F1** | **MCC** | **Recall** | **Specificity** |
| --- | --- | --- | --- | --- | --- | --- | --- |
| +-5%c | SVM | 0.68 | 0.74 | 0.67 | 0.33 | 0.74 | 0.62 |
| +-5%c | Random Forest | 0.65 | 0.77 | 0.64 | 0.34 | 0.64 | 0.64 |
| +-5%c | KNN | 0.69 | 0.69 | 0.59 | 0.32 | 0.64 | 0.74 |
| +-5%c | XGBoost | 0.72 | 0.76 | 0.70 | 0.44 | 0.69 | 0.74 |
| +-5%c | Naive Bayes | 0.67 | 0.73 | 0.67 | 0.40 | 0.74 | 0.60 |
| +-10%c | SVM | 0.74 | 0.80 | 0.73 | 0.47 | **0.75** | 0.72 |
| +-10%c | Random Forest | 0.65 | 0.76 | 0.65 | 0.31 | 0.67 | 0.64 |
| +-10%c | KNN | 0.59 | 0.67 | 0.56 | 0.20 | 0.54 | 0.65 |
| +-10%c | XGBoost | 0.64 | 0.75 | 0.63 | 0.32 | 0.65 | 0.62 |
| +-10%c | Naive Bayes | 0.59 | 0.59 | 0.63 | 0.18 | 0.73 | 0.46 |
| +-15%c | SVM | **0.75** | **0.82** | **0.75** | **0.53** | 0.72 | **0.78** |
| +-15%c | Random Forest | 0.71 | 0.78 | 0.71 | 0.44 | 0.71 | 0.70 |
| +-15%c | KNN | 0.65 | 0.69 | 0.63 | 0.32 | 0.61 | 0.69 |
| +-15%c | XGBoost | 0.71 | 0.79 | 0.71 | 0.46 | 0.69 | 0.73 |
| +-15%c | Naive Bayes | 0.59 | 0.60 | 0.66 | 0.20 | 0.80 | 0.39 |
| +-20%c | SVM | 0.70 | 0.77 | 0.69 | 0.40 | 0.69 | 0.71 |
| +-20%c | Random Forest | 0.66 | 0.73 | 0.66 | 0.33 | 0.66 | 0.66 |
| +-20%c | KNN | 0.56 | 0.63 | 0.53 | 0.12 | 0.50 | 0.62 |
| +-20%c | XGBoost | 0.66 | 0.72 | 0.65 | 0.33 | 0.64 | 0.69 |
| +-20%c | Naive Bayes | 0.59 | 0.59 | 0.66 | 0.19 | 0.80 | 0.37 |
| +-25%c | SVM | 0.68 | 0.74 | 0.68 | 0.36 | 0.67 | 0.69 |
| +-25%c | Random Forest | 0.66 | 0.72 | 0.66 | 0.32 | 0.65 | 0.67 |
| +-25%c | KNN | 0.61 | 0.66 | 0.58 | 0.21 | 0.54 | 0.68 |
| +-25%c | XGBoost | 0.68 | 0.72 | 0.67 | 0.36 | 0.67 | 0.69 |
| +-25%c | Naive Bayes | 0.57 | 0.58 | 0.64 | 0.16 | 0.77 | 0.37 |
| +-30%c | SVM | 0.70 | 0.74 | 0.69 | 0.40 | 0.69 | 0.71 |
| +-30%c | Random Forest | 0.65 | 0.72 | 0.64 | 0.30 | 0.64 | 0.67 |
| +-30%c | KNN | 0.63 | 0.69 | 0.60 | 0.26 | 0.57 | 0.69 |
| +-30%c | XGBoost | 0.66 | 0.73 | 0.64 | 0.32 | 0.64 | 0.68 |
| +-30%c | Naive Bayes | 0.57 | 0.58 | 0.64 | 0.15 | 0.77 | 0.37 |
| +-35%c | SVM | 0.66 | 0.71 | 0.66 | 0.33 | 0.66 | 0.67 |
| +-35%c | Random Forest | 0.65 | 0.70 | 0.65 | 0.31 | 0.64 | 0.67 |
| +-35%c | KNN | 0.61 | 0.67 | 0.59 | 0.23 | 0.56 | 0.66 |
| +-35%c | XGBoost | 0.65 | 0.73 | 0.64 | 0.30 | 0.63 | 0.67 |
| +-35%c | Naive Bayes | 0.56 | 0.57 | 0.64 | 0.14 | 0.78 | 0.34 |
| +-40%c | SVM | 0.63 | 0.70 | 0.63 | 0.26 | 0.62 | 0.64 |
| +-40%c | Random Forest | 0.65 | 0.69 | 0.65 | 0.30 | 0.65 | 0.64 |
| +-40%c | KNN | 0.60 | 0.65 | 0.57 | 0.20 | 0.54 | 0.65 |
| +-40%c | XGBoost | 0.66 | 0.72 | 0.65 | 0.33 | 0.63 | 0.69 |
| +-40%c | Naive Bayes | 0.56 | 0.57 | 0.64 | 0.14 | 0.77 | 0.36 |
| +-45%c | SVM | 0.63 | 0.67 | 0.63 | 0.25 | 0.64 | 0.61 |
| +-45%c | Random Forest | 0.62 | 0.66 | 0.63 | 0.25 | 0.65 | 0.60 |
| +-45%c | KNN | 0.58 | 0.63 | 0.57 | 0.17 | 0.55 | 0.61 |
| +-45%c | XGBoost | 0.64 | 0.67 | 0.63 | 0.27 | 0.62 | 0.65 |
| +-45%c | Naive Bayes | 0.56 | 0.57 | 0.64 | 0.14 | 0.78 | 0.34 |
| +-50%c | SVM | 0.61 | 0.65 | 0.61 | 0.23 | 0.61 | 0.62 |
| +-50%c | Random Forest | 0.62 | 0.66 | 0.63 | 0.25 | 0.64 | 0.61 |
| +-50%c | KNN | 0.60 | 0.62 | 0.58 | 0.20 | 0.57 | 0.63 |
| +-50%c | XGBoost | 0.61 | 0.67 | 0.62 | 0.23 | 0.62 | 0.60 |
| +-50%c | Naive Bayes | 0.56 | 0.56 | 0.63 | 0.12 | 0.78 | 0.34 |
