# Supplementary material for: Predicting drug sensitivity of cancer cells based on DNA methylation levels
Source: PLoS One. 2021 Sep 10;16(9):e0238757. doi: 10.1371/journal.pone.0238757 (PMC8432830; doi:10.1371/journal.pone.0238757)
Supplement: S6 Table — Bold font indicates the best-performing combination for each metric. (DOCX) [file pone.0238757.s021.docx]

| **Scenario** | **Method** | **ACC** | **AUC** | **F1** | **MCC** | **Recall** | **Specificity** |
| --- | --- | --- | --- | --- | --- | --- | --- |
| +-5%c | SVM | 0.58 | NA | 0.50 | 0.22 | 0.55 | 0.60 |
| +-5%c | Random Forest | 0.55 | NA | 0.39 | 0.12 | 0.45 | 0.65 |
| +-5%c | KNN | 0.55 | NA | 0.29 | 0.15 | 0.30 | 0.80 |
| +-5%c | XGBoost | 0.40 | NA | 0.30 | -0.18 | 0.35 | 0.45 |
| +-5%c | Naive Bayes | 0.50 | NA | 0.40 | -0.02 | 0.50 | 0.50 |
| +-10%c | SVM | 0.56 | 0.65 | 0.43 | 0.27 | 0.44 | 0.69 |
| +-10%c | Random Forest | 0.63 | **0.72** | 0.59 | 0.33 | 0.56 | 0.69 |
| +-10%c | KNN | 0.61 | 0.68 | 0.48 | 0.30 | 0.38 | **0.82** |
| +-10%c | XGBoost | 0.55 | 0.58 | 0.52 | 0.18 | 0.49 | 0.62 |
| +-10%c | Naive Bayes | 0.56 | 0.57 | 0.57 | 0.17 | 0.62 | 0.51 |
| +-15%c | SVM | 0.63 | 0.66 | 0.64 | 0.25 | **0.69** | 0.56 |
| +-15%c | Random Forest | **0.70** | 0.70 | **0.68** | **0.41** | 0.64 | 0.76 |
| +-15%c | KNN | 0.62 | 0.61 | 0.56 | 0.24 | 0.51 | 0.75 |
| +-15%c | XGBoost | 0.66 | 0.73 | 0.63 | 0.33 | 0.61 | 0.71 |
| +-15%c | Naive Bayes | 0.59 | 0.59 | 0.59 | 0.18 | 0.61 | 0.58 |
| +-20%c | SVM | 0.59 | 0.66 | 0.55 | 0.19 | 0.54 | 0.63 |
| +-20%c | Random Forest | 0.55 | 0.64 | 0.52 | 0.12 | 0.51 | 0.59 |
| +-20%c | KNN | 0.65 | 0.65 | 0.60 | 0.31 | 0.57 | 0.73 |
| +-20%c | XGBoost | 0.63 | 0.65 | 0.58 | 0.24 | 0.56 | 0.70 |
| +-20%c | Naive Bayes | 0.55 | 0.54 | 0.53 | 0.07 | 0.54 | 0.54 |
| +-25%c | SVM | 0.64 | 0.70 | 0.63 | 0.31 | 0.64 | 0.64 |
| +-25%c | Random Forest | 0.64 | 0.71 | 0.60 | 0.29 | 0.56 | 0.71 |
| +-25%c | KNN | 0.61 | 0.62 | 0.57 | 0.23 | 0.53 | 0.68 |
| +-25%c | XGBoost | 0.62 | 0.67 | 0.57 | 0.25 | 0.54 | 0.69 |
| +-25%c | Naive Bayes | 0.55 | 0.57 | 0.55 | 0.09 | 0.58 | 0.52 |
| +-30%c | SVM | 0.63 | 0.69 | 0.62 | 0.28 | 0.62 | 0.64 |
| +-30%c | Random Forest | 0.62 | 0.70 | 0.59 | 0.24 | 0.55 | 0.68 |
| +-30%c | KNN | 0.62 | 0.68 | 0.59 | 0.23 | 0.57 | 0.68 |
| +-30%c | XGBoost | 0.62 | 0.67 | 0.59 | 0.26 | 0.56 | 0.69 |
| +-30%c | Naive Bayes | 0.60 | 0.61 | 0.61 | 0.19 | 0.65 | 0.55 |
| +-35%c | SVM | 0.62 | 0.72 | 0.60 | 0.28 | 0.56 | 0.69 |
| +-35%c | Random Forest | 0.61 | 0.69 | 0.58 | 0.23 | 0.54 | 0.67 |
| +-35%c | KNN | 0.56 | 0.63 | 0.53 | 0.13 | 0.51 | 0.61 |
| +-35%c | XGBoost | 0.64 | 0.69 | 0.62 | 0.28 | 0.60 | 0.68 |
| +-35%c | Naive Bayes | 0.58 | 0.60 | 0.60 | 0.17 | 0.66 | 0.50 |
| +-40%c | SVM | 0.60 | 0.66 | 0.60 | 0.20 | 0.58 | 0.63 |
| +-40%c | Random Forest | 0.60 | 0.67 | 0.57 | 0.21 | 0.54 | 0.66 |
| +-40%c | KNN | 0.56 | 0.63 | 0.53 | 0.12 | 0.51 | 0.61 |
| +-40%c | XGBoost | 0.62 | 0.68 | 0.60 | 0.24 | 0.57 | 0.66 |
| +-40%c | Naive Bayes | 0.55 | 0.57 | 0.59 | 0.11 | 0.66 | 0.45 |
| +-45%c | SVM | 0.63 | 0.67 | 0.61 | 0.25 | 0.59 | 0.67 |
| +-45%c | Random Forest | 0.61 | 0.68 | 0.59 | 0.22 | 0.56 | 0.65 |
| +-45%c | KNN | 0.63 | 0.66 | 0.60 | 0.25 | 0.56 | 0.69 |
| +-45%c | XGBoost | 0.62 | 0.66 | 0.59 | 0.24 | 0.56 | 0.67 |
| +-45%c | Naive Bayes | 0.58 | 0.59 | 0.60 | 0.18 | 0.64 | 0.52 |
| +-50%c | SVM | 0.62 | 0.68 | 0.60 | 0.24 | 0.56 | 0.68 |
| +-50%c | Random Forest | 0.58 | 0.66 | 0.57 | 0.16 | 0.55 | 0.60 |
| +-50%c | KNN | 0.60 | 0.65 | 0.56 | 0.20 | 0.53 | 0.67 |
| +-50%c | XGBoost | 0.62 | 0.67 | 0.62 | 0.25 | 0.61 | 0.63 |
| +-50%c | Naive Bayes | 0.55 | 0.55 | 0.57 | 0.10 | 0.60 | 0.50 |
