# Supplementary material for: Predicting drug sensitivity of cancer cells based on DNA methylation levels
Source: PLoS One. 2021 Sep 10;16(9):e0238757. doi: 10.1371/journal.pone.0238757 (PMC8432830; doi:10.1371/journal.pone.0238757)
Supplement: S8 Table — Bold font indicates the best-performing combination for each metric. (DOCX) [file pone.0238757.s023.docx]

| **Scenario** | **Method** | **MAE** | **RMSE** | **R^2^** | **Spearman** |
| --- | --- | --- | --- | --- | --- |
| +-5%r | SVM | 1.82 | 2.14 | 0.11 | 0.35 |
| +-5%r | Random Forest | 1.81 | 2.11 | 0.13 | 0.37 |
| +-5%r | KNN | 1.74 | 2.16 | 0.10 | 0.44 |
| +-5%r | XGBoost | 1.60 | 2.16 | 0.07 | 0.43 |
| +-10%r | SVM | 1.26 | 1.59 | 0.37 | 0.49 |
| +-10%r | Random Forest | 1.41 | 1.67 | 0.30 | 0.49 |
| +-10%r | KNN | 1.31 | 1.67 | 0.31 | 0.46 |
| +-10%r | XGBoost | 1.36 | 1.78 | 0.20 | 0.39 |
| +-15%r | SVM | 1.17 | 1.44 | **0.39** | 0.63 |
| +-15%r | Random Forest | 1.29 | 1.52 | 0.32 | 0.56 |
| +-15%r | KNN | 1.30 | 1.62 | 0.23 | 0.45 |
| +-15%r | XGBoost | 1.30 | 1.65 | 0.20 | 0.45 |
| +-20%r | SVM | 1.11 | 1.40 | 0.31 | **0.57** |
| +-20%r | Random Forest | 1.21 | 1.43 | 0.28 | 0.55 |
| +-20%r | KNN | 1.23 | 1.51 | 0.18 | 0.46 |
| +-20%r | XGBoost | 1.21 | 1.48 | 0.23 | 0.47 |
| +-25%r | SVM | 1.08 | 1.33 | 0.24 | 0.52 |
| +-25%r | Random Forest | 1.14 | 1.34 | 0.23 | 0.51 |
| +-25%r | KNN | 1.17 | 1.43 | 0.13 | 0.41 |
| +-25%r | XGBoost | 1.14 | 1.40 | 0.16 | 0.45 |
| +-30%r | SVM | 0.99 | 1.23 | 0.26 | 0.52 |
| +-30%r | Random Forest | 1.06 | 1.26 | 0.24 | 0.51 |
| +-30%r | KNN | 1.10 | 1.34 | 0.13 | 0.40 |
| +-30%r | XGBoost | 1.04 | 1.28 | 0.21 | 0.49 |
| +-35%r | SVM | 0.96 | 1.19 | 0.23 | 0.51 |
| +-35%r | Random Forest | 1.00 | 1.20 | 0.21 | 0.48 |
| +-35%r | KNN | 1.03 | 1.28 | 0.10 | 0.38 |
| +-35%r | XGBoost | 1.01 | 1.24 | 0.16 | 0.43 |
| +-40%r | SVM | 0.92 | 1.15 | 0.17 | 0.46 |
| +-40%r | Random Forest | 0.94 | 1.14 | 0.19 | 0.44 |
| +-40%r | KNN | 0.97 | 1.20 | 0.10 | 0.35 |
| +-40%r | XGBoost | 0.94 | 1.16 | 0.16 | 0.42 |
| +-45%r | SVM | 0.86 | 1.09 | 0.17 | 0.45 |
| +-45%r | Random Forest | 0.87 | 1.08 | 0.19 | 0.44 |
| +-45%r | KNN | 0.91 | 1.14 | 0.09 | 0.33 |
| +-45%r | XGBoost | 0.89 | 1.11 | 0.15 | 0.38 |
| +-50%r | SVM | **0.82** | **1.04** | 0.16 | 0.41 |
| +-50%r | Random Forest | **0.82** | **1.04** | 0.17 | 0.40 |
| +-50%r | KNN | 0.86 | 1.10 | 0.07 | 0.31 |
| +-50%r | XGBoost | **0.82** | 1.05 | 0.15 | 0.38 |
