# Supplementary material for: Predicting drug sensitivity of cancer cells based on DNA methylation levels
Source: PLoS One. 2021 Sep 10;16(9):e0238757. doi: 10.1371/journal.pone.0238757 (PMC8432830; doi:10.1371/journal.pone.0238757)
Supplement: S10 Table — Bold font indicates the best-performing combination for each metric. (DOCX) [file pone.0238757.s025.docx]

| **Scenario** | **Method** | **MAE** | **RMSE** | **R^2^** | **Spearman** |
| --- | --- | --- | --- | --- | --- |
| +-5%r | SVM | 2.83 | 3.17 | 0.06 | 0.39 |
| +-5%r | Random Forest | 3.02 | 3.28 | -0.01 | 0.33 |
| +-5%r | KNN | 2.71 | 3.21 | 0.03 | 0.45 |
| +-5%r | XGBoost | 2.82 | 3.51 | -0.17 | 0.23 |
| +-10%r | SVM | 2.34 | 2.63 | 0.06 | **0.48** |
| +-10%r | Random Forest | 2.53 | 2.75 | 0.00 | 0.37 |
| +-10%r | KNN | 2.33 | 2.74 | -0.02 | 0.41 |
| +-10%r | XGBoost | 2.40 | 2.87 | -0.11 | 0.40 |
| +-15%r | SVM | 2.18 | 2.45 | **0.14** | 0.40 |
| +-15%r | Random Forest | 2.23 | 2.46 | 0.13 | 0.40 |
| +-15%r | KNN | 2.23 | 2.60 | 0.02 | 0.32 |
| +-15%r | XGBoost | 2.25 | 2.61 | 0.01 | 0.29 |
| +-20%r | SVM | 1.96 | 2.25 | 0.08 | 0.40 |
| +-20%r | Random Forest | 2.04 | 2.27 | 0.07 | 0.40 |
| +-20%r | KNN | 1.96 | 2.33 | 0.01 | 0.37 |
| +-20%r | XGBoost | 2.00 | 2.34 | 0.02 | 0.34 |
| +-25%r | SVM | 1.79 | 2.10 | 0.12 | 0.36 |
| +-25%r | Random Forest | 1.84 | 2.08 | 0.13 | 0.36 |
| +-25%r | KNN | 1.82 | 2.21 | 0.03 | 0.29 |
| +-25%r | XGBoost | 1.82 | 2.14 | 0.08 | 0.34 |
| +-30%r | SVM | 1.66 | 1.94 | 0.13 | 0.37 |
| +-30%r | Random Forest | 1.71 | 1.96 | 0.11 | 0.35 |
| +-30%r | KNN | 1.71 | 2.07 | 0.01 | 0.27 |
| +-30%r | XGBoost | 1.72 | 2.04 | 0.05 | 0.29 |
| +-35%r | SVM | 1.54 | 1.83 | 0.11 | 0.34 |
| +-35%r | Random Forest | 1.57 | 1.84 | 0.11 | 0.33 |
| +-35%r | KNN | 1.59 | 1.94 | 0.01 | 0.26 |
| +-35%r | XGBoost | 1.57 | 1.89 | 0.06 | 0.29 |
| +-40%r | SVM | 1.44 | 1.75 | 0.09 | 0.33 |
| +-40%r | Random Forest | 1.47 | 1.76 | 0.08 | 0.30 |
| +-40%r | KNN | 1.49 | 1.83 | -0.01 | 0.26 |
| +-40%r | XGBoost | 1.48 | 1.80 | 0.04 | 0.24 |
| +-45%r | SVM | 1.33 | 1.64 | 0.09 | 0.35 |
| +-45%r | Random Forest | 1.35 | 1.66 | 0.08 | 0.29 |
| +-45%r | KNN | 1.41 | 1.75 | -0.03 | 0.26 |
| +-45%r | XGBoost | 1.37 | 1.70 | 0.02 | 0.24 |
| +-50%r | SVM | **1.24** | 1.59 | 0.06 | 0.30 |
| +-50%r | Random Forest | 1.25 | **1.58** | 0.07 | 0.27 |
| +-50%r | KNN | 1.33 | 1.69 | -0.05 | 0.21 |
| +-50%r | XGBoost | 1.28 | 1.61 | 0.04 | 0.24 |
