# Supplementary material for: Predicting drug sensitivity of cancer cells based on DNA methylation levels
Source: PLoS One. 2021 Sep 10;16(9):e0238757. doi: 10.1371/journal.pone.0238757 (PMC8432830; doi:10.1371/journal.pone.0238757)
Supplement: S11 Table — Bold font indicates the best-performing combination for each metric. (DOCX) [file pone.0238757.s026.docx]

| **Scenario** | **Method** | **MAE** | **RMSE** | **R^2^** | **Spearman** |
| --- | --- | --- | --- | --- | --- |
| +-5%r | SVM | 2.32 | 2.93 | **0.36** | 0.49 |
| +-5%r | Random Forest | 2.36 | 2.93 | **0.36** | 0.49 |
| +-5%r | KNN | 2.29 | 3.03 | 0.32 | 0.43 |
| +-5%r | XGBoost | 2.33 | 3.28 | 0.23 | 0.37 |
| +-10%r | SVM | 2.27 | 2.77 | 0.21 | 0.49 |
| +-10%r | Random Forest | 2.44 | 2.88 | 0.15 | 0.44 |
| +-10%r | KNN | 2.40 | 2.99 | 0.05 | 0.40 |
| +-10%r | XGBoost | 2.50 | 3.24 | -0.09 | 0.29 |
| +-15%r | SVM | 2.21 | 2.62 | 0.27 | **0.53** |
| +-15%r | Random Forest | 2.37 | 2.70 | 0.22 | 0.48 |
| +-15%r | KNN | 2.36 | 2.86 | 0.13 | 0.43 |
| +-15%r | XGBoost | 2.37 | 2.89 | 0.11 | 0.39 |
| +-20%r | SVM | 2.13 | 2.48 | 0.20 | 0.52 |
| +-20%r | Random Forest | 2.28 | 2.57 | 0.14 | 0.45 |
| +-20%r | KNN | 2.28 | 2.71 | 0.03 | 0.41 |
| +-20%r | XGBoost | 2.23 | 2.66 | 0.08 | 0.40 |
| +-25%r | SVM | 1.98 | 2.31 | 0.22 | 0.48 |
| +-25%r | Random Forest | 2.13 | 2.39 | 0.15 | 0.43 |
| +-25%r | KNN | 2.11 | 2.51 | 0.07 | 0.36 |
| +-25%r | XGBoost | 2.04 | 2.39 | 0.15 | 0.42 |
| +-30%r | SVM | 1.90 | 2.20 | 0.17 | 0.44 |
| +-30%r | Random Forest | 2.00 | 2.26 | 0.13 | 0.39 |
| +-30%r | KNN | 2.02 | 2.41 | 0.00 | 0.28 |
| +-30%r | XGBoost | 1.96 | 2.29 | 0.10 | 0.36 |
| +-35%r | SVM | 1.78 | 2.10 | 0.16 | 0.42 |
| +-35%r | Random Forest | 1.86 | 2.14 | 0.13 | 0.36 |
| +-35%r | KNN | 1.87 | 2.25 | 0.04 | 0.29 |
| +-35%r | XGBoost | 1.86 | 2.20 | 0.08 | 0.30 |
| +-40%r | SVM | 1.65 | 1.96 | 0.16 | 0.41 |
| +-40%r | Random Forest | 1.72 | 2.02 | 0.11 | 0.37 |
| +-40%r | KNN | 1.76 | 2.14 | 0.01 | 0.28 |
| +-40%r | XGBoost | 1.69 | 2.01 | 0.12 | 0.37 |
| +-45%r | SVM | 1.52 | 1.86 | 0.17 | 0.43 |
| +-45%r | Random Forest | 1.59 | 1.91 | 0.13 | 0.38 |
| +-45%r | KNN | 1.64 | 2.03 | 0.01 | 0.27 |
| +-45%r | XGBoost | 1.60 | 1.94 | 0.10 | 0.32 |
| +-50%r | SVM | **1.46** | **1.80** | 0.14 | 0.38 |
| +-50%r | Random Forest | 1.49 | 1.84 | 0.09 | 0.32 |
| +-50%r | KNN | 1.56 | 1.94 | -0.01 | 0.24 |
| +-50%r | XGBoost | 1.53 | 1.89 | 0.04 | 0.26 |
