# Supplementary material for: Predicting drug sensitivity of cancer cells based on DNA methylation levels
Source: PLoS One. 2021 Sep 10;16(9):e0238757. doi: 10.1371/journal.pone.0238757 (PMC8432830; doi:10.1371/journal.pone.0238757)
Supplement: S12 Table — Bold font indicates the best-performing combination for each metric. (DOCX) [file pone.0238757.s027.docx]

| **Scenario** | **Method** | **MAE** | **RMSE** | **R^2^** | **Spearman** |
| --- | --- | --- | --- | --- | --- |
| +-5%r | SVM | 3.52 | 4.15 | **0.20** | 0.45 |
| +-5%r | Random Forest | 3.98 | 4.30 | 0.15 | 0.43 |
| +-5%r | KNN | 3.71 | 4.47 | 0.07 | 0.39 |
| +-5%r | XGBoost | 3.79 | 4.79 | -0.04 | 0.41 |
| +-10%r | SVM | 3.35 | 3.94 | 0.09 | 0.45 |
| +-10%r | Random Forest | 3.87 | 4.14 | 0.01 | 0.39 |
| +-10%r | KNN | 3.93 | 4.51 | -0.19 | 0.26 |
| +-10%r | XGBoost | 3.72 | 4.37 | -0.12 | 0.31 |
| +-15%r | SVM | 3.02 | 3.57 | 0.24 | **0.48** |
| +-15%r | Random Forest | 3.39 | 3.71 | 0.18 | 0.42 |
| +-15%r | KNN | 3.45 | 4.07 | 0.01 | 0.33 |
| +-15%r | XGBoost | 3.39 | 4.00 | 0.04 | 0.31 |
| +-20%r | SVM | 3.02 | 3.50 | 0.15 | 0.44 |
| +-20%r | Random Forest | 3.26 | 3.55 | 0.12 | 0.40 |
| +-20%r | KNN | 3.28 | 3.82 | -0.03 | 0.25 |
| +-20%r | XGBoost | 3.18 | 3.71 | 0.03 | 0.30 |
| +-25%r | SVM | 2.81 | 3.25 | 0.17 | 0.43 |
| +-25%r | Random Forest | 3.01 | 3.33 | 0.13 | 0.37 |
| +-25%r | KNN | 2.92 | 3.48 | 0.05 | 0.31 |
| +-25%r | XGBoost | 2.91 | 3.40 | 0.09 | 0.33 |
| +-30%r | SVM | 2.63 | 3.07 | 0.16 | 0.42 |
| +-30%r | Random Forest | 2.73 | 3.09 | 0.15 | 0.38 |
| +-30%r | KNN | 2.72 | 3.28 | 0.05 | 0.31 |
| +-30%r | XGBoost | 2.69 | 3.17 | 0.11 | 0.33 |
| +-35%r | SVM | 2.50 | 2.92 | 0.15 | 0.41 |
| +-35%r | Random Forest | 2.58 | 2.94 | 0.14 | 0.38 |
| +-35%r | KNN | 2.57 | 3.09 | 0.05 | 0.31 |
| +-35%r | XGBoost | 2.57 | 3.02 | 0.08 | 0.31 |
| +-40%r | SVM | 2.36 | 2.77 | 0.13 | 0.40 |
| +-40%r | Random Forest | 2.18 | 2.66 | 0.10 | 0.33 |
| +-40%r | KNN | 2.41 | 2.94 | 0.03 | 0.29 |
| +-40%r | XGBoost | 2.36 | 2.82 | 0.11 | 0.36 |
| +-45%r | SVM | 2.23 | 2.66 | 0.11 | 0.36 |
| +-45%r | Random Forest | 2.17 | 2.61 | 0.10 | 0.34 |
| +-45%r | KNN | 2.28 | 2.82 | 0.00 | 0.27 |
| +-45%r | XGBoost | 2.23 | 2.67 | 0.10 | 0.35 |
| +-50%r | SVM | 2.10 | 2.56 | 0.08 | 0.33 |
| +-50%r | Random Forest | 2.11 | **2.54** | 0.10 | 0.34 |
| +-50%r | KNN | 2.16 | 2.70 | -0.02 | 0.25 |
| +-50%r | XGBoost | **2.08** | **2.54** | 0.09 | 0.33 |
