# Supplementary material for: Predicting drug sensitivity of cancer cells based on DNA methylation levels
Source: PLoS One. 2021 Sep 10;16(9):e0238757. doi: 10.1371/journal.pone.0238757 (PMC8432830; doi:10.1371/journal.pone.0238757)
Supplement: S13 Table — Bold font indicates the best-performing combination for each metric. (DOCX) [file pone.0238757.s028.docx]

| **Scenario** | **Method** | **MAE** | **RMSE** | **R^2^** | **Spearman** |
| --- | --- | --- | --- | --- | --- |
| +-5%r | SVM | 3.27 | 3.56 | -151.71 | 0.08 |
| +-5%r | Random Forest | 3.39 | 3.58 | -134.10 | 0.04 |
| +-5%r | KNN | 3.18 | 3.74 | -111.03 | 0.24 |
| +-5%r | XGBoost | 3.96 | 4.74 | -166.85 | -0.14 |
| +-10%r | SVM | 2.85 | 3.10 | -0.01 | 0.38 |
| +-10%r | Random Forest | 2.95 | 3.11 | -0.02 | 0.30 |
| +-10%r | KNN | 2.84 | 3.36 | -0.18 | **0.39** |
| +-10%r | XGBoost | 2.66 | 3.35 | -0.18 | 0.19 |
| +-15%r | SVM | 2.57 | 2.80 | 0.07 | 0.27 |
| +-15%r | Random Forest | 2.66 | 2.82 | 0.06 | 0.28 |
| +-15%r | KNN | 2.60 | 3.07 | -0.12 | 0.20 |
| +-15%r | XGBoost | 2.71 | 3.26 | -0.26 | 0.13 |
| +-20%r | SVM | 2.40 | 2.67 | 0.01 | 0.24 |
| +-20%r | Random Forest | 2.52 | 2.71 | -0.01 | 0.15 |
| +-20%r | KNN | 2.36 | 2.79 | -0.08 | 0.17 |
| +-20%r | XGBoost | 2.41 | 2.79 | -0.08 | 0.19 |
| +-25%r | SVM | 2.18 | 2.48 | 0.04 | 0.26 |
| +-25%r | Random Forest | 2.25 | 2.47 | 0.04 | 0.26 |
| +-25%r | KNN | 2.18 | 2.60 | -0.06 | 0.22 |
| +-25%r | XGBoost | 2.12 | 2.48 | 0.03 | 0.29 |
| +-30%r | SVM | 2.02 | 2.29 | **0.09** | 0.32 |
| +-30%r | Random Forest | 2.04 | 2.28 | **0.09** | 0.32 |
| +-30%r | KNN | 1.96 | 2.36 | 0.03 | 0.29 |
| +-30%r | XGBoost | 2.05 | 2.40 | 0.00 | 0.24 |
| +-35%r | SVM | 1.85 | 2.18 | 0.05 | 0.34 |
| +-35%r | Random Forest | 1.89 | 2.17 | 0.06 | 0.28 |
| +-35%r | KNN | 1.90 | 2.29 | -0.06 | 0.23 |
| +-35%r | XGBoost | 1.97 | 2.35 | -0.11 | 0.16 |
| +-40%r | SVM | 1.73 | 2.05 | 0.05 | 0.30 |
| +-40%r | Random Forest | 1.75 | 2.04 | 0.05 | 0.31 |
| +-40%r | KNN | 1.76 | 2.15 | -0.04 | 0.22 |
| +-40%r | XGBoost | 1.82 | 2.18 | -0.09 | 0.18 |
| +-45%r | SVM | 1.63 | 1.98 | 0.01 | 0.27 |
| +-45%r | Random Forest | 1.62 | 1.95 | 0.04 | 0.29 |
| +-45%r | KNN | 1.65 | 2.03 | -0.04 | 0.26 |
| +-45%r | XGBoost | 1.65 | 2.03 | -0.02 | 0.24 |
| +-50%r | SVM | **1.49** | 1.87 | 0.04 | 0.29 |
| +-50%r | Random Forest | 1.50 | **1.84** | 0.07 | 0.31 |
| +-50%r | KNN | 1.54 | 1.95 | -0.04 | 0.23 |
| +-50%r | XGBoost | 1.56 | 1.91 | -0.02 | 0.23 |
