# Supplementary material for: Predicting drug sensitivity of cancer cells based on DNA methylation levels
Source: PLoS One. 2021 Sep 10;16(9):e0238757. doi: 10.1371/journal.pone.0238757 (PMC8432830; doi:10.1371/journal.pone.0238757)
Supplement: S14 Table — Bold font indicates the best-performing combination for each metric. (DOCX) [file pone.0238757.s029.docx]

| **Scenario** | **Method** | **MAE** | **RMSE** | **R^2^** | **Spearman** |
| --- | --- | --- | --- | --- | --- |
| +-5%r | SVM | 0.88 | 1.16 | 0.50 | 0.65 |
| +-5%r | Random Forest | 1.06 | 1.28 | 0.40 | 0.61 |
| +-5%r | KNN | 1.03 | 1.35 | 0.33 | 0.65 |
| +-5%r | XGBoost | 1.04 | 1.49 | 0.21 | 0.62 |
| +-10%r | SVM | 0.72 | 0.93 | **0.57** | **0.69** |
| +-10%r | Random Forest | 0.83 | 1.02 | 0.47 | 0.62 |
| +-10%r | KNN | 0.87 | 1.16 | 0.32 | 0.58 |
| +-10%r | XGBoost | 0.82 | 1.11 | 0.37 | 0.62 |
| +-15%r | SVM | 0.66 | 0.86 | 0.56 | **0.69** |
| +-15%r | Random Forest | 0.76 | 0.95 | 0.46 | 0.65 |
| +-15%r | KNN | 0.75 | 1.03 | 0.36 | 0.60 |
| +-15%r | XGBoost | 0.81 | 1.06 | 0.33 | 0.56 |
| +-20%r | SVM | 0.64 | 0.82 | 0.47 | 0.68 |
| +-20%r | Random Forest | 0.73 | 0.90 | 0.37 | 0.62 |
| +-20%r | KNN | 0.73 | 0.97 | 0.27 | 0.52 |
| +-20%r | XGBoost | 0.72 | 0.93 | 0.32 | 0.60 |
| +-25%r | SVM | 0.63 | 0.80 | 0.41 | 0.64 |
| +-25%r | Random Forest | 0.68 | 0.84 | 0.36 | 0.61 |
| +-25%r | KNN | 0.69 | 0.92 | 0.24 | 0.53 |
| +-25%r | XGBoost | 0.70 | 0.88 | 0.29 | 0.54 |
| +-30%r | SVM | 0.63 | 0.80 | 0.34 | 0.59 |
| +-30%r | Random Forest | 0.63 | 0.79 | 0.37 | 0.60 |
| +-30%r | KNN | 0.67 | 0.88 | 0.22 | 0.50 |
| +-30%r | XGBoost | 0.67 | 0.84 | 0.29 | 0.54 |
| +-35%r | SVM | 0.60 | 0.76 | 0.32 | 0.55 |
| +-35%r | Random Forest | 0.61 | 0.76 | 0.31 | 0.56 |
| +-35%r | KNN | 0.65 | 0.84 | 0.15 | 0.47 |
| +-35%r | XGBoost | 0.63 | 0.81 | 0.22 | 0.51 |
| +-40%r | SVM | 0.57 | 0.72 | 0.31 | 0.55 |
| +-40%r | Random Forest | 0.57 | 0.73 | 0.29 | 0.53 |
| +-40%r | KNN | 0.63 | 0.81 | 0.08 | 0.45 |
| +-40%r | XGBoost | 0.60 | 0.78 | 0.17 | 0.49 |
| +-45%r | SVM | 0.54 | 0.69 | 0.29 | 0.53 |
| +-45%r | Random Forest | 0.54 | 0.70 | 0.27 | 0.51 |
| +-45%r | KNN | 0.59 | 0.78 | 0.07 | 0.42 |
| +-45%r | XGBoost | 0.56 | 0.73 | 0.19 | 0.49 |
| +-50%r | SVM | 0.52 | 0.68 | 0.22 | 0.50 |
| +-50%r | Random Forest | **0.51** | **0.67** | 0.26 | 0.49 |
| +-50%r | KNN | 0.55 | 0.73 | 0.10 | 0.41 |
| +-50%r | XGBoost | 0.53 | 0.69 | 0.21 | 0.48 |
