# Supplementary material for: Predicting drug sensitivity of cancer cells based on DNA methylation levels
Source: PLoS One. 2021 Sep 10;16(9):e0238757. doi: 10.1371/journal.pone.0238757 (PMC8432830; doi:10.1371/journal.pone.0238757)
Supplement: S15 Table — We used the feature selection to identify informative genes for Cisplatin drug-response prediction. Genomic coordinates are based on build 37 of the human genome. We used information gain to rank the genes; a higher score indicates a more informative gene. (DOCX) [file pone.0238757.s030.docx]

| **Classification** | | | **Regression** | | |
| --- | --- | --- | --- | --- | --- |
| *Gene* | *Coordinates* | *Score* | *Gene* | *Coordinates* | *Score* |
| CGN | chr1:151483573-151483902 | 0.251 | C17orf81, CLDN7 | chr17:7164285-7166245 | 0.069 |
| CLDN4, WBSCR27 | chr7:73245434-73246045 | 0.207 | CLDN4, WBSCR27 | chr7:73245434-73246045 | 0.066 |
| JMJD6, MXRA7 | chr17:74706465-74707067 | 0.199 | CLDN3 | chr7:73183379-73185115 | 0.059 |
| MYO5C | chr15:52587353-52588172 | 0.196 | ESRP1 | chr8:95652455-95652873 | 0.056 |
| C17orf81, CLDN7 | chr17:7164285-7166245 | 0.195 | CDH1 | chr16:68771034-68772344 | 0.055 |
| FUT2 | chr19:49206443-49206818 | 0.188 | TUBGCP2, ZNF511 | chr10:135123238-135123448 | 0.051 |
| ID3 | chr1:23885682-23886212 | 0.187 | IFT172, KRTCAP3, NRBP1 | chr2:27664939-27665151 | 0.051 |
| EFR3A | chr8:132916322-132917060 | 0.186 | LAD1 | chr1:201368560-201369032 | 0.049 |
| LOC100129354, NBEAL2 | chr3:47050486-47051609 | 0.185 | TUBGCP2, ZNF511 | chr10:135122851-135123109 | 0.047 |
| AKR1B1 | chr7:134143115-134144063 | 0.183 | SPINT1 | chr15:41135719-41137210 | 0.046 |
| TUBGCP2, ZNF511 | chr10:135122851-135123109 | 0.177 | HRC | chr19:49655102-49655395 | 0.045 |
| BRD3 | chr9:136919143-136919376 | 0.175 | SYK | chr9:93563775-93564546 | 0.044 |
| KIRREL2, NPHS1 | chr19:36347044-36348101 | 0.174 | C1orf172 | chr1:27286065-27287101 | 0.043 |
| FKBP2, VEGFB | chr11:64008283-64009487 | 0.173 | CGN | chr1:151483573-151483902 | 0.043 |
| BASP1 | chr5:17275369-17275638 | 0.171 | ESRP1 | chr8:95653898-95654733 | 0.043 |
| CMTM3 | chr16:66638254-66639561 | 0.171 | LLGL2, TSEN54 | chr17:73520956-73522540 | 0.043 |
| CCDC19 | chr1:159869901-159870143 | 0.170 | RAB4A | chr1:229406646-229407129 | 0.042 |
| ITGA5 | chr12:54811981-54812202 | 0.170 | MYO5C | chr15:52587353-52588172 | 0.042 |
| VIM | chr10:17270430-17272617 | 0.169 | MAP7 | chr6:136870826-136872145 | 0.041 |
| EPS8L2, TMEM80 | chr11:705794-706534 | 0.169 | CDC42BPG | chr11:64611714-64612634 | 0.041 |
