# Supplementary material for: Predicting drug sensitivity of cancer cells based on DNA methylation levels
Source: PLoS One. 2021 Sep 10;16(9):e0238757. doi: 10.1371/journal.pone.0238757 (PMC8432830; doi:10.1371/journal.pone.0238757)
Supplement: S16 Table — We used the feature selection to identify informative genes for Docetaxel drug-response prediction. Genomic coordinates are based on build 37 of the human genome. We used information gain to rank the genes; a higher score indicates a more informative gene. (DOCX) [file pone.0238757.s031.docx]

| **Classification** | | | **Regression** | | |
| --- | --- | --- | --- | --- | --- |
| *Gene* | *Coordinates* | *Score* | *Gene* | *Coordinates* | *Score* |
| ELK3 | chr12:96588665-96589145 | 0.357 | NFATC2 | chr20:50158904-50159509 | 0.086 |
| DAPK3 | chr19:3970536-3970746 | 0.327 | VGLL4 | chr3:11610137-11610370 | 0.083 |
| SNAI2 | chr8:49835987-49836231 | 0.302 | CSNK1E | chr22:38712684-38713333 | 0.073 |
| EXT1 | chr8:119123974-119124432 | 0.296 | COL7A1, UQCRC1 | chr3:48631882-48632901 | 0.069 |
| VGLL4 | chr3:11610137-11610370 | 0.288 | FLRT2 | chr14:85996494-85996958 | 0.068 |
| MMP14, MRPL52 | chr14:23305893-23307013 | 0.268 | C8orf58, PDLIM2 | chr8:22456091-22456508 | 0.068 |
| NCOR2 | chr12:125003217-125003482 | 0.264 | DAPK3 | chr19:3970536-3970746 | 0.067 |
| CMAH | chr6:25139920-25140246 | 0.261 | PLEKHG5 | chr1:6545143-6545559 | 0.063 |
| PRNP | chr20:4666827-4667874 | 0.253 | EMP3 | chr19:48833394-48833720 | 0.063 |
| PLEKHG5 | chr1:6550083-6551115 | 0.250 | RAB34 | chr17:27044168-27045049 | 0.062 |
| DUSP5 | chr10:112257163-112258684 | 0.248 | C22orf9, MIR1249 | chr22:45598721-45599080 | 0.060 |
| CBR3 | chr21:37507198-37508259 | 0.240 | ELK3 | chr12:96588665-96589145 | 0.060 |
| TNK2 | chr3:195622187-195623033 | 0.240 | PIK3CG | chr7:106508057-106508733 | 0.059 |
| GADD45A | chr1:68150913-68152270 | 0.236 | PTRF | chr17:40573740-40575526 | 0.059 |
| FLRT2 | chr14:85996494-85996958 | 0.232 | EIF3G | chr19:10230162-10230682 | 0.059 |
| ZC3H7B | chr22:41697388-41698601 | 0.232 | ERBB2 | chr17:37856448-37856891 | 0.059 |
| EIF3G | chr19:10230162-10230682 | 0.231 | HCFC1R1, THOC6, TNFRSF12A | chr16:3073686-3074443 | 0.058 |
| GPR176 | chr15:40211961-40213444 | 0.228 | SOLH | chr16:587567-588172 | 0.056 |
| COL7A1, UQCRC1 | chr3:48631882-48632901 | 0.228 | INPP5D | chr2:233925091-233925318 | 0.056 |
| PTK2 | chr8:142010440-142011907 | 0.220 | COG5, DUS4L | chr7:107204114-107204797 | 0.056 |
