# Supplementary material for: Predicting drug sensitivity of cancer cells based on DNA methylation levels
Source: PLoS One. 2021 Sep 10;16(9):e0238757. doi: 10.1371/journal.pone.0238757 (PMC8432830; doi:10.1371/journal.pone.0238757)
Supplement: S17 Table — We used the feature selection to identify informative genes for Doxorubicin drug-response prediction. Genomic coordinates are based on build 37 of the human genome. We used information gain to rank the genes; a higher score indicates a more informative gene. (DOCX) [file pone.0238757.s032.docx]

| **Classification** | | | **Regression** | | |
| --- | --- | --- | --- | --- | --- |
| *Gene* | *Coordinates* | *Score* | *Gene* | *Coordinates* | *Score* |
| SKAP1 | chr17:46507344-46507778 | 0.195 | TMEM177 | chr2:120436530-120437010 | 0.033 |
| SLC27A2 | chr15:50474322-50475186 | 0.194 | NEK10 | chr3:27410612-27411066 | 0.031 |
| PYGM, RASGRP2 | chr11:64509433-64513826 | 0.188 | ZFP3 | chr17:4981357-4981979 | 0.028 |
| CGN, MIR554, TUFT1 | chr1:151512661-151513199 | 0.184 | WDYHV1 | chr8:124428605-124429425 | 0.027 |
| LACTB2, XKR9 | chr8:71581050-71581650 | 0.181 | PPM1H | chr12:63328143-63329135 | 0.027 |
| MXRA8 | chr1:1289707-1291126 | 0.173 | NCRNA00029 | chr20:61665780-61666555 | 0.027 |
| CAMK2N1 | chr1:20810462-20813511 | 0.173 | MATN2 | chr8:98881311-98881843 | 0.026 |
| OSTC | chr4:109571693-109572039 | 0.173 | RIMKLA | chr1:42845978-42846988 | 0.026 |
| PTK2 | chr8:142010440-142011907 | 0.169 | INHBB | chr2:121101800-121104534 | 0.026 |
| CGN | chr1:151483573-151483902 | 0.167 | GDA | chr9:74764241-74764903 | 0.026 |
| SCIN | chr7:12610165-12610834 | 0.166 | CMAS | chr12:22199062-22199589 | 0.025 |
| C2orf43 | chr2:21022564-21022934 | 0.162 | ATP1B2 | chr17:7554139-7555338 | 0.025 |
| TMEM45B | chr11:129685737-129686211 | 0.155 | C3orf57 | chr3:161089626-161090649 | 0.025 |
| TMEM177 | chr2:120436530-120437010 | 0.153 | C8orf84 | chr8:74005021-74005856 | 0.024 |
| RG9MTD3 | chr9:37753655-37753949 | 0.152 | STYXL1, TMEM120A | chr7:75623357-75624164 | 0.024 |
| CLDN4, WBSCR27 | chr7:73245434-73246045 | 0.151 | ICA1 | chr7:8301031-8302252 | 0.024 |
| GAL | chr11:68451359-68452846 | 0.150 | YBX2 | chr17:7197431-7198417 | 0.024 |
| CGB7 | chr19:49559222-49560497 | 0.150 | TUBGCP2, ZNF511 | chr10:135123238-135123448 | 0.024 |
| COMMD2 | chr3:149469909-149470388 | 0.150 | CP110, GDE1 | chr16:19535074-19535635 | 0.024 |
| PODXL | chr7:131242693-131243006 | 0.150 | TMEM219 | chr16:29973023-29973570 | 0.024 |
