# Supplementary material for: Predicting drug sensitivity of cancer cells based on DNA methylation levels
Source: PLoS One. 2021 Sep 10;16(9):e0238757. doi: 10.1371/journal.pone.0238757 (PMC8432830; doi:10.1371/journal.pone.0238757)
Supplement: S18 Table — We used the feature selection to identify informative genes for Etoposide drug-response prediction. Genomic coordinates are based on build 37 of the human genome. We used information gain to rank the genes; a higher score indicates a more informative gene. (DOCX) [file pone.0238757.s033.docx]

| **Classification** | | | **Regression** | | |
| --- | --- | --- | --- | --- | --- |
| *Gene* | *Coordinates* | *Score* | *Gene* | *Coordinates* | *Score* |
| GNMT, PEX6 | chr6:42928218-42928810 | 0.367 | C8orf84 | chr8:74005021-74005856 | 0.041 |
| TRUB1 | chr10:116697893-116698376 | 0.314 | RBP4 | chr10:95360389-95361387 | 0.039 |
| RHPN2 | chr19:33555246-33556431 | 0.308 | AQP11 | chr11:77300360-77301391 | 0.036 |
| USP43, WDR16 | chr17:9548389-9549616 | 0.303 | TBR1 | chr2:162270888-162271413 | 0.035 |
| ARHGAP21 | chr10:25011963-25013816 | 0.301 | ANKRD37, UFSP2 | chr4:186317143-186318255 | 0.035 |
| HOOK1 | chr1:60280624-60281048 | 0.300 | LACTB2, XKR9 | chr8:71581050-71581650 | 0.034 |
| ANKRD13D, SSH3 | chr11:67070807-67071801 | 0.299 | PCCA | chr13:100740956-100741805 | 0.034 |
| SLC44A2 | chr19:10735999-10736396 | 0.290 | FAM111B | chr11:58873889-58874486 | 0.034 |
| SPRY4 | chr5:141705391-141705688 | 0.290 | CGN, MIR554, TUFT1 | chr1:151512661-151513199 | 0.033 |
| DDAH1 | chr1:85929940-85931168 | 0.288 | C5orf49 | chr5:7850957-7851413 | 0.033 |
| RHOU | chr1:228870810-228872297 | 0.288 | TJP1 | chr15:30114110-30115215 | 0.033 |
| MARVELD3 | chr16:71659829-71660747 | 0.282 | DNAJC5, TPD52L2 | chr20:62525796-62526638 | 0.033 |
| MOSC2 | chr1:220921411-220922176 | 0.279 | RNF20 | chr9:104295917-104296232 | 0.032 |
| IFT88 | chr13:21140951-21141719 | 0.279 | EPB41L4B | chr9:112083333-112083549 | 0.032 |
| KRT18 | chr12:53342805-53343162 | 0.278 | EPS8 | chr12:15941718-15942740 | 0.032 |
| CYB5A | chr18:71958141-71959770 | 0.276 | GNMT, PEX6 | chr6:42928218-42928810 | 0.032 |
| CRB3, DENND1C | chr19:6463991-6464780 | 0.276 | CYP39A1, SLC25A27 | chr6:46620541-46621189 | 0.031 |
| EPS8 | chr12:15941718-15942740 | 0.275 | LOC646762 | chr7:29724188-29725436 | 0.031 |
| TMEM171 | chr5:72415611-72416766 | 0.274 | RHEB | chr7:151216068-151217901 | 0.031 |
| ADCY6 | chr12:49183049-49183282 | 0.271 | TMEM45B | chr11:129685737-129686211 | 0.031 |
