# Supplementary material for: Predicting drug sensitivity of cancer cells based on DNA methylation levels
Source: PLoS One. 2021 Sep 10;16(9):e0238757. doi: 10.1371/journal.pone.0238757 (PMC8432830; doi:10.1371/journal.pone.0238757)
Supplement: S19 Table — We used the feature selection to identify informative genes for Gemcitabine drug-response prediction. Genomic coordinates are based on build 37 of the human genome. We used information gain to rank the genes; a higher score indicates a more informative gene. (DOCX) [file pone.0238757.s034.docx]

| **Classification** | | | **Regression** | | |
| --- | --- | --- | --- | --- | --- |
| *Gene* | *Coordinates* | *Score* | *Gene* | *Coordinates* | *Score* |
| IFFO1, NOP2 | chr12:6664425-6665336 | 0.220 | YBX2 | chr17:7197431-7198417 | 0.033 |
| LOC100287834 | chr7:62858468-62858826 | 0.216 | TMEM177 | chr2:120436530-120437010 | 0.032 |
| TNK1 | chr17:7284223-7284687 | 0.203 | MACROD2 | chr20:13976700-13977068 | 0.032 |
| RNF39 | chr6:30042918-30043500 | 0.199 | ZNF793 | chr19:37997790-37998125 | 0.032 |
| C1orf229 | chr1:247274585-247275757 | 0.190 | CCDC64 | chr12:120426547-120428066 | 0.030 |
| SLC44A2 | chr19:10735999-10736396 | 0.185 | DUSP8, HCCA2, LOC338651 | chr11:1593550-1594378 | 0.030 |
| FAM174B | chr15:93198374-93199181 | 0.184 | NEK10 | chr3:27410612-27411066 | 0.029 |
| EFNA1 | chr1:155098434-155100451 | 0.183 | CHN1 | chr2:175869574-175870289 | 0.029 |
| LAD1 | chr1:201368560-201369032 | 0.183 | ATP6V1C2 | chr2:10861206-10862382 | 0.029 |
| BIRC8 | chr19:53794411-53794732 | 0.182 | TLR2 | chr4:154605086-154606052 | 0.029 |
| YBX2 | chr17:7197431-7198417 | 0.179 | ZNF514 | chr2:95824802-95825721 | 0.028 |
| CRB3, DENND1C | chr19:6463991-6464780 | 0.179 | CA8 | chr8:61193312-61194195 | 0.028 |
| KIAA0284 | chr14:105332408-105332651 | 0.178 | C17orf81, CLDN7 | chr17:7164285-7166245 | 0.027 |
| LOC100287704, LOC100287834 | chr7:62809609-62809812 | 0.178 | SCAI | chr9:127905675-127905947 | 0.027 |
| CFDP1 | chr16:75466850-75467527 | 0.176 | MANSC1 | chr12:12502942-12503465 | 0.027 |
| C11orf90 | chr11:93583374-93583717 | 0.172 | SHC2 | chr19:457800-462256 | 0.027 |
| CYR61, DDAH1 | chr1:86046362-86047240 | 0.166 | STK25 | chr2:242447017-242448558 | 0.027 |
| TEAD4 | chr12:3067960-3069444 | 0.166 | ACPL2 | chr3:140951193-140951451 | 0.027 |
| CAMK2G | chr10:75633600-75634796 | 0.162 | ZFP3 | chr17:4981357-4981979 | 0.027 |
| HM13 | chr20:30102057-30102856 | 0.162 | RUFY1 | chr5:178986513-178986999 | 0.027 |
