# Supplementary material for: Predicting drug sensitivity of cancer cells based on DNA methylation levels
Source: PLoS One. 2021 Sep 10;16(9):e0238757. doi: 10.1371/journal.pone.0238757 (PMC8432830; doi:10.1371/journal.pone.0238757)
Supplement: S20 Table — We used the feature selection to identify informative genes for Paclitaxel drug-response prediction. Genomic coordinates are based on build 37 of the human genome. We used information gain to rank the genes; a higher score indicates a more informative gene. (DOCX) [file pone.0238757.s035.docx]

| **Classification** | | | **Regression** | | |
| --- | --- | --- | --- | --- | --- |
| *Gene* | *Coordinates* | *Score* | *Gene* | *Coordinates* | *Score* |
| C2orf29 | chr2:101869023-101869876 | 0.347 | TUBB2B | chr6:3227025-3229688 | 0.062 |
| LMO2 | chr11:33890357-33891495 | 0.342 | PRDM6 | chr5:122424905-122425958 | 0.059 |
| BLOC1S1, ITGA7, RDH5 | chr12:56109798-56110298 | 0.315 | ONECUT3 | chr19:1753216-1755606 | 0.059 |
| CTNNA2 | chr2:79739696-79740243 | 0.307 | DGKA | chr12:56325774-56326223 | 0.056 |
| DTNA | chr18:32073444-32074292 | 0.274 | PNMAL1 | chr19:46974557-46975073 | 0.056 |
| CDC40, WASF1 | chr6:110500025-110500966 | 0.274 | AP1S1, VGF | chr7:100806279-100809064 | 0.054 |
| HMGA1 | chr6:34202567-34206193 | 0.274 | CNTNAP2 | chr7:145813030-145814084 | 0.054 |
| PHYHIPL | chr10:60935827-60937049 | 0.244 | ZFP36 | chr19:39897241-39898942 | 0.054 |
| GALNTL6 | chr4:172733734-172735118 | 0.244 | SLC10A4 | chr4:48485362-48486473 | 0.054 |
| ZNF625 | chr19:12266998-12267686 | 0.244 | PAPOLB, RADIL | chr7:4901336-4901753 | 0.053 |
| ATP5J, GABPA | chr21:27106814-27108211 | 0.238 | TMEM25, TTC36 | chr11:118401235-118402069 | 0.053 |
| psiTPTE22 | chr22:17083384-17083628 | 0.238 | ABCB1 | chr7:87230059-87230260 | 0.053 |
| CACNG8 | chr19:54466357-54466725 | 0.230 | NOL4 | chr18:31802358-31803792 | 0.053 |
| FBXO36, TRIP12 | chr2:230785912-230787665 | 0.224 | DLL3 | chr19:39989397-39990140 | 0.052 |
| EYA4 | chr6:133562086-133563586 | 0.224 | MCFD2, TTC7A | chr2:47167858-47168978 | 0.052 |
| EBPL | chr13:50265224-50265598 | 0.224 | B3GAT1 | chr11:134257428-134257631 | 0.052 |
| DPRXP4, RNF135 | chr17:29298046-29298606 | 0.224 | ADAMTS3 | chr4:73434855-73435321 | 0.051 |
| AIM1 | chr6:106959764-106960985 | 0.224 | MAP3K12 | chr12:53886562-53887101 | 0.051 |
| IMMP2L | chr7:111202079-111202683 | 0.224 | C6orf97 | chr6:151814980-151815527 | 0.051 |
| SFPQ | chr1:35657467-35658811 | 0.216 | PIK3R3 | chr1:46598126-46599129 | 0.051 |
