# Supplementary material for: Predicting drug sensitivity of cancer cells based on DNA methylation levels
Source: PLoS One. 2021 Sep 10;16(9):e0238757. doi: 10.1371/journal.pone.0238757 (PMC8432830; doi:10.1371/journal.pone.0238757)
Supplement: S21 Table — We used the feature selection to identify informative genes for Temozolomide drug-response prediction. Genomic coordinates are based on build 37 of the human genome. We used information gain to rank the genes; a higher score indicates a more informative gene. (DOCX) [file pone.0238757.s036.docx]

| **Classification** | | | **Regression** | | |
| --- | --- | --- | --- | --- | --- |
| *Gene* | *Coordinates* | *Score* | *Gene* | *Coordinates* | *Score* |
| TJP1 | chr15:30114110-30115215 | 0.345 | NELF, PNPLA7 | chr9:140356314-140356987 | 0.087 |
| AGAP2, LOC100130776 | chr12:58119909-58121551 | 0.329 | TJP1 | chr15:30114110-30115215 | 0.085 |
| ARHGAP29 | chr1:94702690-94703344 | 0.316 | MGAT1 | chr5:180229375-180230147 | 0.082 |
| C4orf14, POLR2B | chr4:57842634-57843893 | 0.307 | PLEKHA1 | chr10:124134088-124134933 | 0.082 |
| RARA | chr17:38472958-38473201 | 0.298 | DDAH1 | chr1:85929940-85931168 | 0.081 |
| ZNF280D | chr15:57025347-57026150 | 0.295 | TEAD1 | chr11:12695414-12696981 | 0.081 |
| SYDE1 | chr19:15217951-15218617 | 0.283 | DSTN | chr20:17549628-17550051 | 0.079 |
| TUBGCP2, ZNF511 | chr10:135122851-135123109 | 0.281 | ICAM3, RAVER1 | chr19:10443688-10446022 | 0.077 |
| ACP1, SH3YL1 | chr2:263400-265238 | 0.277 | SLC44A2 | chr19:10735999-10736396 | 0.076 |
| TBC1D12 | chr10:96162023-96163327 | 0.275 | CHST12 | chr7:2442792-2444011 | 0.075 |
| CTU1 | chr19:51607207-51607840 | 0.274 | FERMT3, STIP1 | chr11:63974829-63975048 | 0.075 |
| LARGE | chr22:34315841-34318637 | 0.272 | GAS2L3 | chr12:100967293-100967845 | 0.075 |
| UTRN | chr6:144605926-144608280 | 0.269 | CASZ1 | chr1:10853894-10856964 | 0.074 |
| AK1 | chr9:130639738-130640143 | 0.269 | SPN | chr16:29675845-29676120 | 0.074 |
| DOCK1 | chr10:128593609-128595048 | 0.269 | PTPN14 | chr1:214724104-214725056 | 0.074 |
| NSUN7 | chr4:40751842-40752493 | 0.267 | LOC100133985 | chr2:70352204-70352531 | 0.073 |
| PARD6G | chr18:78004028-78005438 | 0.264 | ERRFI1 | chr1:8085554-8086854 | 0.073 |
| RRN3P2 | chr16:29086220-29086434 | 0.262 | TMEM149, U2AF1L4 | chr19:36231186-36232219 | 0.073 |
| PKN1 | chr19:14551998-14552255 | 0.259 | FAT1 | chr4:187644319-187648253 | 0.072 |
| AGAP2 | chr12:58132478-58132734 | 0.259 | GNG7 | chr19:2578956-2579746 | 0.072 |
