# Supplementary material for: Predicting drug sensitivity of cancer cells based on DNA methylation levels
Source: PLoS One. 2021 Sep 10;16(9):e0238757. doi: 10.1371/journal.pone.0238757 (PMC8432830; doi:10.1371/journal.pone.0238757)
Supplement: S22 Table — We used a statistical overrepresentation test to identify protein classes associated with the top-20 ranked genes in the feature-selection analysis. (DOCX) [file pone.0238757.s037.docx]

| **Gefitinib** | | | | |
| --- | --- | --- | --- | --- |
| *Gene Set Name*  *[# Genes (K)]* | *Description* | *# Genes in Overlap (k)* | *p-value* | *FDR q-value* |
| KOINUMA_TARGETS_OF_SMAD2_OR_SMAD3  [843] | Genes with promoters occupied by SMAD2 or SMAD3 [GeneID=4087, 4088] in HaCaT cells (keratinocyte) according to a ChIP-chip analysis. | 9 | 1.81 e-10 | 1.32 e-6 |
| GENTILE_UV_RESPONSE_CLUSTER_D4 [54] | Cluster d4: genes progressively down-regulated in WS1 cells (fibroblast) through 12 h after irradiation with high dose UV-C. | 3 | 3.02 e-6 | 1.1 e-2 |
| FRIDMAN_SENESCENCE_UP [77] | Genes up-regulated in senescent cells. | 3 | 8.85 e-6 | 2.15 e-2 |
| SHEDDEN_LUNG_  CANCER_GOOD_  SURVIVAL_A12 [320] | Cluster 12 of method A: up-regulation of these genes in patients with non-small cell lung cancer (NSCLC) predicts good survival outcome. | 4 | 2.15 e-5 | 3.9 e-2 |
| TSUNODA_CISPLATIN_  RESISTANCE_UP [15] | Genes up-regulated in bladder cancer cells resistant to cisplatin [PubChem=2767] compared to the parental cells sensitive to the drug. | 2 | 2.74 e-5 | 3.98 e-2 |
|  | | | | |
| **Cisplatin** | | | | |
| *Gene Set Name*  *[# Genes (K)]* | *Description* | *# Genes in Overlap (k)* | *p-value* | *FDR q-value* |
| CHARAFE_BREAST_  CANCER_LUMINAL_VS_MESEN_SENCHYMAL_  DN [465] | Genes down-regulated in luminal-like breast cancer cell lines compared to the mesenchymal-like ones. | 6 | 7.17 e-7 | 4.94 e-3 |
| KOINUMA_TARGETS_OF_SMAD2_OR_SMAD3  [843] | Genes with promoters occupied by SMAD2 or SMAD3 [GeneID=4087, 4088] in HaCaT cells (keratinocyte) according to a ChIP-chip analysis. | 7 | 1.43 e-6 | 4.94 e-3 |
| REACTOME_CELL_CELL_COMMUNICATION [130] | Cell-Cell communication | 4 | 2.04 e-6 | 4.94 e-3 |
| SENGUPTA_  NASOPHARYNGEAL_  CARCINOMA_DN [358] | Genes down-regulated in nsopharyngeal carcinoma relative to the normal tissue. | 5 | 4.59 e-6 | 8.35 e-3 |
| HUPER_BREAST_BASAL_VS_LUMINAL_DN [58] | Genes down-regulated in basal mammary epithelial cells compared to the luminal ones. | 3 | 9.19 e-6 | 1.34 e-2 |
| GU_PDEF_TARGETS_UP  [71] | Integrin, VEGF, Wnt and TGFbeta signaling pathway genes up-regulated in PC-3 cells (prostate cancer) after knockdown of PDEF [GeneID=25803] by RNAi. | 3 | 1.69 e-5 | 1.55 e-2 |
| ONDER_CDH1_TARGETS_2_DN [473] | Genes down-regulated in HMLE cells (immortalized nontransformed mammary epithelium) after E-cadhedrin (CDH1) [GeneID=999] knockdown by RNAi. | 5 | 1.76 e-5 | 1.55 e-2 |
| COLDREN_GEFITINIB_  RESISTANCE_DN [228] | Genes down-regulated in NSCLC (non-small cell lung carcinoma) cell lines resistant to gefitinib [PubChem=123631] compared to the sensitive ones. | 4 | 1.88 e-5 | 1.55 e-2 |
| WP_PRIMARY_FOCAL_  SEGMENTAL_  GLOMERULOSC_  OSCLEROSIS_FSGS [74] | Primary Focal Segmental Glomerulosclerosis FSGS | 3 | 1.92 e-5 | 1.55 e-2 |
| FERRANDO_T_ALL_  WITH_MLL_ENL_  FUSION_UP [89] | Top 100 genes positively associated with T-cell acute lymphoblastic leukemia MLL T-ALL) expressing MLL-ENL fusion [GeneID=4297;4298]. | 3 | 3.33 e-5 | 2.23 e-2 |
|  | | | | |
| **Docetaxel** | | | | |
| *Gene Set Name*  *[# Genes (K)]* | *Description* | *# Genes in Overlap (k)* | *p-value* | *FDR q-value* |
| CHARAFE_BREAST_  CANCER_LUMINAL_VS_MESEN_SENCHYMAL_  DN [465] | Genes down-regulated in luminal-like breast cancer cell lines compared to the mesenchymal-like ones. | 7 | 3.97 e-9 | 1.82 e-5 |
| LIM_MAMMARY_  STEM_CELL_UP [481] | Genes consistently up-regulated in mammary stem cells both in mouse and human species. | 7 | 5.02 e-9 | 1.82 e-5 |
| HUANG_DASATINIB_  RESISTANCE_UP [80] | Genes whose expression positively correlated with sensitivity of breast cancer cell lines to dasatinib [PubChem=3062316]. | 4 | 1.05 e-7 | 2.54 e-4 |
| KOINUMA_TARGETS_OF_SMAD2_OR_SMAD3  [843] | Genes with promoters occupied by SMAD2 or SMAD3 [GeneID=4087, 4088] in HaCaT cells (keratinocyte) according to a ChIP-chip analysis. | 7 | 2.3 e-7 | 4.19 e-4 |
| PETROVA_  ENDOTHELIUM_  LYMPHATIC_VS_BLOOD_OOD_DN [162] | Genes down-regulated in BEC (blood endothelial cells) compared to LEC (lymphatic endothelial cells). | 4 | 1.78 e-6 | 2.59 e-3 |
| SESTO_RESPONSE_TO_UV_C5 [46] | Cluster 5: genes changed in primary keratinocytes by UVB irradiation. | 3 | 2.15 e-6 | 2.6 e-3 |
| EGFR_UP.V1_UP [192] | Genes up-regulated in MCF-7 cells (breast cancer) positive for ESR1 [Gene ID=2099] and engineered to express ligand-activatable EGFR [Gene ID=1956]. | 4 | 3.49 e-6 | 3.35 e-3 |
| MITSIADES_RESPONSE_  TO_APLIDIN_UP [446] | Genes up-regulated in the MM1S cells (multiple myeloma) after treatment with aplidin [PubChem=44152164], a marine-derived compound with potential anti-cancer properties. | 5 | 3.76 e-6 | 3.35 e-3 |
| CHARAFE_BREAST_  CANCER_LUMINAL_VS_  BASAL_SAL_DN [455] | Genes down-regulated in luminal-like breast cancer cell lines compared to the basal-like ones. | 5 | 4.15 e-6 | 3.35 e-3 |
| ENK_UV_RESPONSE_  EPIDERMIS_DN [513] | Genes down-regulated in epidermis after to UVB irradiation. | 5 | 7.42 e-6 | 5.4 e-3 |
|  | | | | |
| **Doxorubicin** | | | | |
| *Gene Set Name*  *[# Genes (K)]* | *Description* | *# Genes in Overlap (k)* | *p-value* | *FDR q-value* |
| CHARAFE_BREAST_  CANCER_LUMINAL_VS_  MESEN_SENCHYMAL_  UP [453] | Genes up-regulated in luminal-like breast cancer cell lines compared to the mesenchymal-like ones. | 5 | 6.43 e-6 | 2.61 e-2 |
| PILON_KLF1_TARGETS_  UP [501] | Genes up-regulated in erythroid progenitor cells from fetal livers of E13.5 embryos with KLF1 [GeneID=10661] knockout compared to those from the wild type embryos. | 5 | 1.05 e-5 | 2.61 e-2 |
| DUTERTRE_ESTRADIOL_RESPONSE_24HR_  DN [504] | Genes down-regulated in MCF7 cells (breast cancer) at 24 h of estradiol [PubChem=5757] treatment. | 5 | 1.08 e-5 | 2.61 e-2 |
| MIKKELSEN_MEF_HCP_  WITH_H3K27ME3 [590] | Genes with high-CpG-density promoters (HCP) bearing histone H3 trimethylation mark at K27 (H3K27me3) in MEF cells (embryonic fibroblast). | 5 | 2.29 e-5 | 4.17 e-2 |
|  | | | | |
| **Etoposide** | | | | |
| *Gene Set Name*  *[# Genes (K)]* | *Description* | *# Genes in Overlap (k)* | *p-value* | *FDR q-value* |
| CHARAFE_BREAST_  CANCER_LUMINAL_  VS_MESEN_  SENCHYMAL_UP [453] | Genes up-regulated in luminal-like breast cancer cell lines compared to the mesenchymal-like ones. | 6 | 2.29 e-7 | 1.66 e-3 |
| MODULE_180 [119] | Genes in the cancer module 180. | 4 | 7.5 e-7 | 2.73 e-3 |
| LIM_MAMMARY_  STEM_CELL_DN [416] | Genes consistently down-regulated in mammary stem cells both in mouse and human species. | 5 | 4.25 e-6 | 1.03 e-2 |
| MODULE_342 [213] | Genes in the cancer module 342. | 4 | 7.59 e-6 | 1.38 e-2 |
| MEISSNER_NPC_HCP_  WITH_H3_  UNMETHYLATED [542] | Genes with high-CpG-density promoters (HCP) that have no histone H3 methylation marks in neural precursor cells (NPC). | 5 | 1.53 e-5 | 1.96 e-2 |
| BOYLAN_MULTIPLE_MYELOMA_D_DN [82] | Genes down-regulated in group D of tumors arising from overexpression of BCL2L1 and MYC [GeneID=598;4609] in plasma cells. | 3 | 1.62 e-5 | 1.96 e-2 |
| MEISSNER_BRAIN_HCP_WITH_H3K27ME3 [271] | Genes with high-CpG-density promoters (HCP) bearing the H3K27 tri-methylation (H3K27me3) mark in brain. | 4 | 1.95 e-5 | 2.03 e-2 |
|  | | | | |
| **Gemcitabine** | | | | |
| No overlaps found. | | | | |
|  | | | | |
| **Paclitaxel** | | | | |
| No overlaps found. | | | | |
|  | | | | |
| **Temozolomide** | | | | |
| No overlaps found. | | | | |
