# Supplementary material for: Predicting drug sensitivity of cancer cells based on DNA methylation levels
Source: PLoS One. 2021 Sep 10;16(9):e0238757. doi: 10.1371/journal.pone.0238757 (PMC8432830; doi:10.1371/journal.pone.0238757)
Supplement: S23 Table — We used a statistical overrepresentation test to identify protein classes associated with the top-20 ranked genes in the feature-selection analysis. (DOCX) [file pone.0238757.s038.docx]

| **Gefitinib** | | | | |
| --- | --- | --- | --- | --- |
| *Gene Set Name*  *[# Genes (K)]* | *Description* | *# Genes in Overlap (k)* | *p-value* | *FDR q-value* |
| RICKMAN_TUMOR_  DIFFERENTIATED_WELL_  VS_PS_POORLY_DN [380] | Down-regulated genes that vary between HNSCC (head and neck squamous cell carcinoma) groups formed on the basis of their level of pathological differentiation: well vs poorly differentiated tumors. | 5 | 3.4 e-6 | 1.9 e-2 |
|  |  |  |  |  |
| EGFR_UP.V1_UP  [192] | Genes up-regulated in MCF-7 cells (breast cancer) positive for ESR1 [Gene ID=2099] and engineered to express ligand-activatable EGFR [Gene ID=1956]. | 4 | 5.97 e-6 | 1.9 e-2 |
| COLLER_MYC_  TARGETS_DN [7] | Genes down-regulated in 293T (transformed fetal renal cell) upon expression of MYC [GeneID=4609]. | 2 | 7.83 e-6 | 1.9 e-2 |
|  | | | | |
| **Cisplatin** | | | | |
| *Gene Set Name*  *[# Genes (K)]* | *Description* | *# Genes in Overlap (k)* | *p-value* | *FDR q-value* |
| HOLLERN_EMT_BREAST_  TUMOR_DN [123] | Genes that that have low expression in mammary tumors of epithelial-mesenchymal transition (EMT) histology. | 9 | 2.26 e-17 | 1.64 e-13 |
| CHARAFE_BREAST_  CANCER_LUMINAL_VS_  MESEN_SENCHYMAL_  UP [453] | Genes up-regulated in luminal-like breast cancer cell lines compared to the mesenchymal-like ones. | 11 | 7.47 e-16 | 2.72 e-12 |
|  |  |  |  |  |
| ONDER_CDH1_TARGETS_  2_DN [473] | Genes down-regulated in HMLE cells (immortalized nontransformed mammary epithelium) after E-cadhedrin (CDH1) [GeneID=999] knockdown by RNAi. | 9 | 4.61 e-12 | 1.12 e-8 |
| COLDREN_GEFITINIB_  RESISTANCE_DN [228] | Genes down-regulated in NSCLC (non-small cell lung carcinoma) cell lines resistant to gefitinib [PubChem=123631] compared to the sensitive ones. | 7 | 5.61 e-11 | 1.02 e-7 |
| MCBRYAN_PUBERTAL_  BREAST_4_5WK_UP [270] | Genes up-regulated during pubertal mammary gland development between week 4 and 5. | 7 | 1.83 e-10 | 2.66 e-7 |
| WU_CELL_MIGRATION  [183] | Genes associated with migration rate of 40 human bladder cancer cells. | 6 | 1.05 e-9 | 1.27 e-6 |
| LIM_MAMMARY_STEM_  CELL_DN  [416] | Genes consistently down-regulated in mammary stem cells both in mouse and human species. | 7 | 3.67 e-9 | 3.82 e-6 |
| BOYAULT_LIVER_CANCER_  SUBCLASS_G1_UP  [116] | Up-regulated genes in hepatocellular carcinoma (HCC) subclass G1, defined by unsupervised clustering. | 5 | 7.58 e-9 | 6.89 e-6 |
| MODULE_180 [119] | Genes in the cancer module 180. | 5 | 8.62 e-9 | 6.97 e-6 |
| KEGG_TIGHT_  JUNCTION [132] | Tight junction. | 5 | 1.45 e-8 | 1.06 e-5 |
|  | | | | |
| **Docetaxel** | | | | |
| *Gene Set Name*  *[# Genes (K)]* | *Description* | *# Genes in Overlap (k)* | *p-value* | *FDR q-value* |
| NIKOLSKY_BREAST_  CANCER_16P13_  AMPLICON [119] | Genes within amplicon 16p13 identified in a study of 191 breast tumor samples. | 4 | 1.05 e-6 | 7.64 e-3 |
| CHARAFE_BREAST_  CANCER_LUMINAL_VS_  MESEN_SENCHYMAL_  DN [465] | Genes down-regulated in luminal-like breast cancer cell lines compared to the mesenchymal-like ones. | 5 | 1.11 e-5 | 2.89 e-2 |
|  |  |  |  |  |
| KOINUMA_TARGETS_OF_  SMAD2_OR_SMAD3 [843] | Genes with promoters occupied by SMAD2 or SMAD3 [GeneID=4087, 4088] in HaCaT cells (keratinocyte) according to a ChIP-chip analysis. | 6 | 1.37 e-5 | 2.89 e-2 |
| KEGG_B_CELL_RECEPTOR_SIGNALING_PATHWAY [75] | B cell receptor signaling pathway | 3 | 1.59 e-5 | 2.89 e-2 |
|  | | | | |
| **Doxorubicin** | | | | |
| No overlaps found. | | | | |
|  | | | | |
| **Etoposide** | | | | |
| *Gene Set Name*  *[# Genes (K)]* | *Description* | *# Genes in Overlap (k)* | *p-value* | *FDR q-value* |
| SENGUPTA_NASOPHARYNGEAL_CARCINOMA_DN  [358] | Genes down-regulated in nsopharyngeal carcinoma relative to the normal tissue. | 5 | 3.12 e-6 | 2.27 e-2 |
|  | | | | |
| **Gemcitabine** | | | | |
| No overlaps found. | | | | |
|  | | | | |
| **Paclitaxel** | | | | |
| No overlaps found. | | | | |
|  | | | | |
| **Temozolomide** | | | | |
| No overlaps found. | | | | |
