# Supplementary material for: Predicting drug sensitivity of cancer cells based on DNA methylation levels
Source: PLoS One. 2021 Sep 10;16(9):e0238757. doi: 10.1371/journal.pone.0238757 (PMC8432830; doi:10.1371/journal.pone.0238757)
Supplement: Attachment — Submitted filename: Response to reviewers.pdf [file pone.0238757.s040.pdf]

Response to reviewers' comments

Response to Reviewer #1:

(1) This paper does not discuss much on the related work of using machine learning models to predict drug sensitivity. The classification and regression models used in this paper are classic methods. What are the state-of-art methods for predicting drug sensitivity of cancer cells?

Reply: We thank the reviewer for providing a helpful and thorough review. We have expanded our coverage of existing literature regarding the use of machine-learning algorithms for predicting drug sensitivity. In our study, we used classical algorithms as a way to establish a performance baseline against which other algorithms might be compared when predicting drug responses based on DNA methylation data. Furthermore, our study focused primarily on evaluating the potential to subsample relatively extreme response values rather than on introducing new algorithms. We now include the following paragraph in the Introduction section:

"Many computational methods have been proposed to predict anticancer drug sensitivity based on genetic, genomic, or epigenomic features of cancer cell lines. The most common approach is to generate a drug-specific model, which is independently trained using molecular observations and drug-response data from cell lines tested with each drug individually. Linear-regression based, drug-specific models have been developed using gene expression data (Barretina et al., 2012; Geeleher et al., 2014; Iorio et al., 2016) or a combination of gene expression data and other genomic data types, such as copy number alterations and DNA methylation (Chen and Sun, 2017). Non-linear models using a single data type or multiple data types have also been proposed, including artificial neural networks, random forests, support vector machines, kernel regression, latent and Bayesian approaches, attractor landscape analysis of network dynamics, unsupervised pathway activity models, and recommender systems (Costello et al., 2014; Dong et al., 2015; Zhang et al., 2015; Ammad-ud-din et al., 2016; Corte's-Ciriano et al., 2016; Gupta et al., 2016; Ammad-ud-din et al., 2017; Choi et al., 2017; Rahman et al., 2017; Ali et al., 2018; Chang et al., 2018; Dhruba et al., 2018; Ding et al. 2018; Huang et al., 2018; Suphavilai et al., 2018; Wang et al., 2019; Xu et al., 2019; Emdadi et al., 2020). Transfer-learning techniques have also been proposed to improve drug-response prediction performance for one type of cancer by incorporating data from other types of cancer (Turki et al., 2017). Drug response information has also been modeled in combination with chemical drug properties using elastic net regression, support vector machines, regularized matrix factorization, and manifold Learning (Menden et al., 2013; Yuan et al., 2016; Wang et al., 2017; Moughari et al., 2020; Su et al., 2020)."

(2) In the section regression results, it shows the results for different scenarios, such as +-5%r, +10%r etc. I understand that the scenario in classification (e.g., +-5%c) corresponds to different thresholds to convert continuous outputs to discrete outputs. However, why we need different scenarios in regression task.

Reply: We agree that subsampling makes more sense for classification tasks than for regression tasks. However, we determined that it would be valuable to evaluate subsampling for both types of tasks, in part because an underlying motivation of this approach was to evaluate the potential to decrease data-generation costs. For example, if it could be shown that generating data for relatively few (extreme) responders performs as well as or better than generating data for responders across the full range of response values, it could lead to cost savings. We have added comments to the Introduction and Discussion sections that address these issues.

(3) In table 8, it shows the informative genes for predicting cell-line response for Gefitinib. It seems that the top-ranked genes for classification task are totally different to regression task. How to explain it? The data and the objective seems the same. The difference is that one formulate it as a classification task and the other formulate it as a regression task.

Reply: This is an important question. Although the general objectives and data were the same for classification and regression—and algorithms (e.g., Random Forests, Support Vector Machines) often use similar methodologies for classification and regression—it is not unlikely that different genes would be selected for classification versus regression. Firstly, we had data for thousands of genes, and different genes may exhibit similar methylation patterns, so the algorithms may choose different (correlated) genes by random chance. Secondly, the algorithms optimized against different objective functions for classification versus regression; even small differences in how the algorithms prioritized genes could lead to considerable differences in the gene ranks. We have added a comment about this in the Results section to provide additional perspective for the reader.

(4) More detailed discussion may be needed for the conclusion "classification models derived from cell-line data failed to generalize effectively for tumors".

Reply: We thank the reviewer for highlighting this need. We have expanded this part of the Discussion section.

Response to Reviewer #2:

(1) The main findings should be highlighted in the abstract and the conclusion sections.

Reply: We thank the reviewer for bringing this to our attention. We have revised and expanded these sections according to the reviewer's suggestion.

(2) The lineages in cell lines were overlooked in modeling frameworks and interpretations.

Reply: We agree that this was a limitation of our study. Systematic differences surely exist across different cell-line lineages. As we designed this study, we preferred that our analysis combine all available cell-line data in GDSC as a way to increase the overall sample size and identify patterns that may span lineages. However, in response to your comment and a comment from another reviewer, we have modified our analysis by adjusting the data for cell type (and data source) using an empirical Bayesian framework. As with the original analysis,

this approach has some limitations. For example, there were as few as 2-3 samples available for some of the cell types. We address this issue in the Discussion now. Regardless, as shown in our updated manuscript, the results were similar using this approach.

(*3) The GDSC cell lines were measured on the multi-omic scale, DNA, mRNA and protein. In the introduction section, clearer motivation evaluating only DNA methylation is required.*

Reply: Thank you for this suggestion. We now provide more justification for this in the manuscript.

(4) A number of classification algorithms for discretized drug response, and regression approaches for continuous IC50 were evaluated in terms of the predictive accuracy. However evaluation between the two types seems to be missing.

Reply: Thank you for this suggestion. We have used the Spearman correlation coefficient as a way to assess the concordance between the predicted probabilities (classification algorithms) and predicted IC50 values (regression algorithms). We have added a graph that illustrates the similarities and differences between these types of predictions.

(5) Detailed description on feature selection should be included. CTGF gene and etc were highlighted in the paper. Were these appeared using all the algorithms? What was the estimated prediction accuracy based on the algorithms? Did these genes show the high predictive power for all the drugs and all the cell line lineages?

Reply: We performed feature selection in a *post hoc* analysis after we performed classification. Accordingly, we did not compare the ability to predict drug responses with and without feature selection. We had multiple reasons for this: 1) we were concerned about overfitting the training data, which we used to tune hyperparameters for classification algorithms, and 2) additional tuning would have been required to select the best *n* features to use for each classification scenario. To clarify these points, we have changed the terminology that we use in the paper from "feature selection" to "feature ranking." In addition, we have clarified in the manuscript that we used information gain to rank the features, have provided an explanation of the intuition behind information gain, and have clarified that the feature-ranking analysis was performed *post hoc*. The Supplementary Material provides the feature ranks and scores for all of the drugs and algorithms.

(*6) For patients' drug response, the four categories response call was used. When you evaluate the algorithms on the patient data, what was the response call for the cell lines based on IC50? And how do you train the models in the cell lines data? Greater detail is required.*

Reply: Thank you for letting us know that this description was not clear enough. We now provide more details. After training the classification algorithms on the cell-line dataset to discriminate between low and high IC50 responses, we used the algorithms to generate probabilistic predictions for each tumor sample. We then evaluated whether the probabilistic predictions of treatment response were correlated with the reported responses for each patient as a way to assess whether higher probabilities of response (based on cell-line

observations) correlated with better patient responses. The latter approach is slightly different than we described in the original manuscript. We modified our methodology based on a recommendation from one of the other reviewers (see below).

Response to Reviewer #3:

(1) The introduction lacks an appropriate literature review. It is better to review some papers that have used machine learning methods for predicting drug response. Moreover, it is required to present a summary of other features that have been used, such as mutation, copy number variation, and the papers that have used a combination of several types. For example:
1) Moughari, Fatemeh Ahmadi, and Changiz Eslahchi. "ADRML: anticancer drug response prediction using manifold learning." Scientific Reports 10, no. 1 (2020): 1-18.
2) Emdadi, Akram, and Changiz Eslahchi. "DSPLMF: A Method for Cancer Drug Sensitivity Prediction Using a Novel Regularization Approach in Logistic Matrix Factorization." Frontiers in Genetics 11 (2020): 75.
3) Xu, Xiaolu, Hong Gu, Yang Wang, Jia Wang, and Pan Qin. "Autoencoder based feature selection method for classification of anticancer drug response." Frontiers in genetics 10 (2019): 233.
4) Chang, Yoosup, Hyejin Park, Hyun-Jin Yang, Seungju Lee, Kwee-Yum Lee, Tae Soon Kim, Jongsun Jung, and Jae-Min Shin. "Cancer drug response profile scan (CDRscan): a deep learning model that predicts drug effectiveness from cancer genomic signature." Scientific Reports 8, no. 1 (2018): 1-11.
And many more papers.

Reply: We thank the reviewer for this suggestion. We have expanded our coverage of existing literature regarding the use of machine-learning algorithms for predicting drug sensitivity. We now include the following paragraph in the Introduction section:

"Many computational methods have been proposed to predict anticancer drug sensitivity based on genetic, genomic, or epigenomic features of cancer cell lines. The most common approach is to generate a drug-specific model, which is independently trained using molecular observations and drug-response data from cell lines tested with each drug individually. Linear-regression based, drug-specific models have been developed using gene expression data (Barretina et al., 2012; Geeleher et al., 2014; Iorio et al., 2016) or a combination of gene expression data and other genomic data types, such as copy number alterations and DNA methylation (Chen and Sun, 2017). Non-linear models using a single data type or multiple data types have also been proposed, including artificial neural networks, random forests, support vector machines, kernel regression, latent and Bayesian approaches, attractor landscape analysis of network dynamics, unsupervised pathway activity models, and recommender systems (Costello et al., 2014; Dong et al., 2015; Zhang et al., 2015; Ammad-ud-din et al., 2016; Corte's-Ciriano et al., 2016; Gupta et al., 2016; Ammad-ud-din et al., 2017; Choi et al., 2017; Rahman et al., 2017; Ali et al., 2018; Chang et al., 2018; Dhruba et al., 2018; Ding et al. 2018; Huang et al., 2018; Suphavilai et al., 2018; Wang et al., 2019; Xu et al., 2019; Emdadi et al., 2020). Transfer-learning techniques have also been proposed to improve drug-response prediction performance for one type of

cancer by incorporating data from other types of cancer (Turki et al., 2017). Drug response information has also been modeled in combination with chemical drug properties using elastic net regression, support vector machines, regularized matrix factorization, and manifold Learning (Menden et al., 2013; Yuan et al., 2016; Wang et al., 2017; Moughari et al., 2020; Su et al., 2020)."

(2) The method used for feature selection and identify essential genes is not described well. It is required to give a brief description and intuitive about the method used in this section. Relying on solely naming the used packages is not sufficient, and readers may have no clue of what is going on in this analysis.

Reply: We have added more details about the feature-selection approach that we used in the Methods section, including a description of the intuition behind information gain.

(3) The authors have conducted two biological analyses (1- finding important genes 2- predicting drug response for TCGA tumors). However, both analyzes do not yield significant results. The genes found as important genes for predicting anticancer drug response do not play a significant role in cancer procedures. Moreover, both the regression and classification methods were unable to predict drug response for TCGA tumors. Hence, biological analysis does not confirm model competence. It is required to conduct a more significant biological analysis.

Reply: We agree that our methodology did not result in strong predictive performance on the tumor data in most cases or extensive insights into the biology of drug responses (based on the top-ranked genes and subsequent functional analyses). However, our study does provide valid scientific insights, including about subsampling, which was the main focus of our study. Furthermore, even negative results provide value to the scientific community. As the PLOS One policy about negative results states, "In keeping with our mission to publish all valid research, we consider negative and null results."

(4) The authors do not have a significant contribution to the method proposing. They analyze the off-the-shelf machine learning methods in various scenarios.

Reply: The main focus of this research was to study the effect of subsampling on model performance rather than to contribute new algorithms to the field. We evaluated the subsampling strategy using traditional machine learning algorithms as a way to estimate the overall potential to predict drug responses from methylation-based cell-line data. Future studies could explore the potential to improve predictive performance with alternative algorithms or data types.

(5) Authors have used DNA methylation data instead of gene expression because gene expression data is not stable and is measured using various technologies. It is required to use another type of feature using the same model to determine DNA methylation features' effectiveness.

Reply: We have expanded our coverage of prior literature, including studies that have been conducted using other data types. However, the focus of our study was not to evaluate or

compare the predictive accuracy of multiple data types but rather to estimate the overall potential to predict drug responses from methylation-based cell-line data and to evaluate the effects of data subsampling.

(6) The Enrichment of informative genes in GSEA cancer-related gene families can better illustrate the influence of informative genes in cancer-related procedures. Moreover, finding protein complexes related to these genes using CORUM and iRefWeb websites may reveal more information about the drug mechanism.

Reply: We thank the reviewer for these suggestions. We modified the enrichment analysis to use genes sets (including many which are cancer relation) from the Molecular Signatures Database (also used by GSEA) and their gene-set overlap method. This provided much more relevant results than the previous analysis, including insights about drug resistance.

(7) The regression analysis to show the correlation of predicted continuous probabilities with ordinal TCGA drug response labels seems inappropriate. The authors can use hypothesis testings that are specifically designed for ordinal variables.

Reply: Thank you for this suggestion. After looking at this again, we realized that our approach was flawed. We have modified our approach to use the Spearman rank correlation coefficient as a measure of the similarities between the predicted probabilities and the ordinal responses for TCGA patients. Spearman's method is applicable to ordinal values (see Lehman, Ann (2005). Jmp For Basic Univariate And Multivariate Statistics: A Step-by-step Guide. Cary, NC: SAS Press. p. 123. ISBN 978-1-59047-576-8.). We calculated p-values for these comparisons and adjusted for multiple tests.

(8) Moreover, it is better to apply a pre-processing method to remove batch effects.

Reply: Thank you for this suggestion. In response to this comment, we have performed a type of batch adjustment. Although batch information was not available at a granular level, we used data source as a batch variable and performed batch-effect adjustment using an Empirical Bayes approach. We also included cell type as a covariate to help mitigate the effects of that variable (per a suggestion from another reviewer). We repeated the entire analysis using these data and have updated these results in the manuscript. Our conclusions remain essentially identical; however, we agree that this resulted in a less-biased dataset.

(9) The authors have conducted their analyses on only eight specific drugs. Therefore, the analyses are not comprehensive and may have biased results. It is better to incorporate many more drugs in analyses or select eight randomly drugs.

Reply: We selected these drugs based on the amount of available data. With the exception of Gefitinib, these drugs were those that overlapped most between the GDSC and TCGA datasets. We included Gefitinib in our final analysis because it is well known and because we used it in our initial testing. However, we selected the remaining drugs based on data availability. Although it would be ideal to use additional drugs and select them at random, we concluded that selecting drugs based on sample size would make the best use of available data.

(10) The computed criteria for regression methods is not very diverse. RMSE is just the square root of the MSE. Assessing MSE beside RMSE does not convey additional information. Moreover, MAE has almost the same perspective of vision for model evaluation. It is better to compute more diverse criteria such as Pearson correlation coefficient or R^2.

Reply: Thank you for this suggestion. We have excluded MSE and included R^2 and the Spearman rank correlation coefficient in the regression analyses.

Response to Reviewer #4:

(1) Table 1: adding in the supplementary a brief description of the hyperparameters selected for each model could help understanding better the experimental setup. For example the Cost in the SVM is probably referring to the regularization parameter in the cost function, but it is not clear for a reader with limited machine learning knowledge.

Reply: We thank the reviewer for highlighting this need. We have modified this table by adding a brief description of each parameter.

(2) Figures, in general, can benefit from an improved quality, mainly resolution (Figure 2,3). Figure 4 bigger markers and fonts. Also a common style in terms of font type, size and color scheme.

Reply: In our revised manuscript, we have provided high-resolution (TIFF) versions of our figures. In addition, we have modified Figure 4 (now Figure 5) so that it is taller and thus easier to read.

(3) Regarding the regression analysis it would be very interesting to see reported also the correlation values, to have a better idea on the trend of the predictions beyond the average (MSE, RMSE and MAE).

Reply: Thank you for this suggestion. We have excluded MSE (as RMSE is the square root of the MSE) and have included R^2 and the Spearman rank correlation coefficient in the regression analyses.

(4) This might be a bit beyond the scope of the paper, but as shown, in Costello et al. (https://www.nature.com/articles/nbt.2877.pdf), the expression values (transcriptomic) seem to be more predictive compared to other omic features for the different predictive models considered. I think it would be interesting, if it is not too much work, to repeat the translational analysis with transcriptomic data, to see whether the poor performance observed at methylomic level are consistent or change with a different data type.

Reply: We agree that comparing our results with those obtained using transcriptomic data would be an interesting comparison; we thank the reviewer for this suggestion. One reason

we used methylation data is that data were collected using the same technology (Illumina HumanMethylation 450K arrays) for GDSC as well as TCGA. However, GDSC used a relatively obscure type of gene-expression microarray (Affymetrix U219), whereas TCGA used RNA-Sequencing. Although these types of data can be combined for some types of analyses, we concluded that it would be challenging to correct the datasets properly for these platform effects. In other words, the machine-learning algorithms could easily be "distracted" by these platform differences. Furthermore, the main focus of this work was to study the effects of subsampling on model performance, and we were able to address that question using methylation data.

Response to Reviewer #5:

(1) Page 3
Line 11: Different platform and preprocess algorithms were considered challenging points in combining gene expression datasets. I believe it is also the case in general for combining methylation datasets even though GDSC and TCGA used the same methylation profiling assays.

Reply: We thank the reviewer for this observation. We agree that biases can arise even though two datasets were generated using the same platform. However, one reason we chose methylation data was that the data had been preprocessed using the same methodology for GDSC and TCGA. In contrast, the gene-expression data were prepared using gene-expression microarrays (Affymetrix A219) or RNA-Sequencing (RNA-Sequencing), thus resulting in much more pronounced differences between the platforms. In addition, in response to a comment from one of the other reviewers, we have performed a type of batch adjustment in which we corrected for differences between the datasets (as well as cell type). Our updated manuscript includes these results, which are qualitatively similar to the previous ones.

(2) Page 11
Line 3-6: Performances of various classification algorithms used in this study were well summarized in Table 2 by the proportions of cell lines used for training classifiers of cell lines' responses to Gemcitabine. However, there is no description nor information about how many methylation loci (or regions) were used in classification and regression analyses.

Reply: Thank you for letting us know that this was not clear. We have modified the Methods section to clarify that we used all available, gene-level summarized values as input to the classification and regression algorithms.

(3) Page 15
To choose the best combination of classification algorithm and the proportion of cell cell lines, average AUC ranks across drugs were calculated. It would be informative to readers to present the standard deviation and range of observed AUCs along with the AUC ranks in Table 3.

Reply: Thank you for this suggestion. Standard deviation and ranges of observed AUC values have been added to Table 3. Standard deviation and ranges of observed RMSE values have been added to Table 6.

(4) Page 26 and 27.
To examine the association of classification probability with patients' outcomes, authors used a linear regression. It would be better to display mean and standard deviation of the probability. For an association test, it is suggested to compare means of the probability between four outcome groups.

Reply: Thank you for your comment. Perhaps we misunderstand the reviewer's comment, but we did not have a way to calculate a mean or standard deviation for the probabilities because we trained only once (per drug) and then made one prediction per patient. Each algorithm produces a single set of probabilistic predictions per patient. One of the other reviewers suggested that we use an approach for evaluating the probabilities that accounts for the ordinal nature of the patient drug responses. We modified this part of the analysis to use the Spearman rank correlation coefficient.

(5) Table S24: I have a question about Table S24. Is the sample size n in the table the number of cell lines or the number of cancer patients? Please clarify it and I also recommend presenting both numbers in this table.

Reply: The *n* value indicates the number of cancer patients for a specific tissue and drug. We have clarified this in the table caption.

(6) Page 30
Line 16-18: Authors reported in the result section that using a small (5-10%) of cancer cell lines with extreme IC50s yields the best performance in training a classification model while all samples did in training a regression model. It would be meaningful if you can discuss the reason or insight for this observation because the subsampling of cancer cell lines is the primary focus of this study.

Reply: We thank the reviewer for highlighting that subsampling was the primary focus of this study. In response to this comment and comments from other reviewers, we have modified the Discussion section to address the differences we observed between classification and regression in more depth. We have also added an analysis in which we compare the classification results directly against the regression results using a common metric.
