# Supplementary material for: Predicting drug sensitivity of cancer cells based on DNA methylation levels
Source: PLoS One. 2021 Sep 10;16(9):e0238757. doi: 10.1371/journal.pone.0238757 (PMC8432830; doi:10.1371/journal.pone.0238757)
Supplement: Attachment — Submitted filename: PONE-D-20-26313_reviewer.docx [file pone.0238757.s039.docx]

**Manuscript Number PONE-D-20-26313
"Predicting drug sensitivity of cancer cells based on DNA methylation levels"**

The authors evaluated machine learning algorithms in predicting drug sensitivity in cell lines using DNA methylation. Although extensive amount of work has been done, the key points and main conclusion of the work are vague, and the impact of this study is doubtful.

1. The main findings should be highlighted in the abstract and the conclusion sections.
2. The lineages in cell lines were overlooked in modeling frameworks and interpretations.
3. The GDSC cell lines were measured on the multi-omic scale, DNA, mRNA and protein. In the introduction section, clearer motivation evaluating only DNA methylation is required.
4. A number of classification algorithms for discretized drug response, and regression approaches for continuous IC50 were evaluated in terms of the predictive accuracy. However evaluation between the two types seems to be missing.
5. Detailed description on feature selection should be included. CTGF gene and etc were highlighted in the paper. Were these appeared using all the algorithms? What was the estimated prediction accuracy based on the algorithms? Did these genes show the high predictive power for all the drugs and all the cell line lineages?
6. For patients’ drug response, the four categories response call was used. When you evaluate the algorithms on the patient data, what was the response call for the cell lines based on IC50? And how do you train the models in the cell lines data? Greater detail is required.
